# Solid Wettability Modification via Adsorption of Antimicrobial Sucrose Fatty Acid Esters and Some Other Sugar-Based Surfactants

**DOI:** 10.3390/molecules23071597

**Published:** 2018-07-01

**Authors:** Joanna Krawczyk

**Affiliations:** Department of Interfacial Phenomena, Faculty of Chemistry, Maria Curie-Skłodowska University, Maria Curie-Skłodowska Sq. 3, 20-031 Lublin, Poland; j.krawczyk@poczta.umcs.lublin.pl; Tel.: +48 (81) 537-56-03

**Keywords:** sucrose fatty acids esters, sugar surfactants, polymers, quartz, adhesion, wettability

## Abstract

Solid–liquid interface properties play a crucial role in the adsorption and adhesion of different microorganisms to the solid. There are some methods to inhibit microorganisms’ adsorption at the solid–liquid interface and their adhesion to the solid. These methods can be divided into bulk phase and surface modification. They are often based on the surfactants’ effect on the wettability of the solid in a given system, due to the fact that adsorption and wetting properties of the food additive antimicrobial surfactants (sucrose monolaurate and sucrose monodecanoate as well as some other sugar-based ones (*n*-octyl-*β*-d-glucopyranoside, *n*-dodecyl-*β*-d-glucopyranoside, *n*-dodecyl-*β*-d-maltoside)) in the solid-aqueous solution of surfactant-air system were considered. Quantitative description of adsorption of the studied compounds at the solid–liquid interface was made based on the contact angle of the aqueous solutions of studied surfactants on polytetrafluoroethylene, polyethylene, poly(methyl methacrylate), polyamide and quartz surface and their surface tension. From the above-mentioned considerations, it can be seen that during the wettability process of the studied solids, surfactants are oriented in a specific direction depending on the type of the solid and surfactant. This specific orientation and adsorption of surfactant molecules at the solid–water interface cause changes of the solid surface properties and its wettability, which was successfully predicted in the studied systems.

## 1. Introduction

Wettability of solids by different liquids or solutions plays a crucial role in many industries, medicine, pharmacy and everyday life [1,2,3]. It is usually evaluated by indirect means since surface and interfacial tension of a solid cannot be easily measured directly. One of these methods involves measuring the contact angle (θ) of a given liquid or solution being at equilibrium with the other two phases (gas and solid substrate) with which it contacts.

From a practical point of view, aqueous solutions are the most common; however, because of its high surface tension water does not spontaneously spread over most solid surfaces (polymers or minerals) [1]. Wetting properties of water or aqueous solutions can be changed by the addition of surface active agents (surfactants) which, due to their amphiphilic structure, are able to adsorb at different interfaces and change their properties. Adsorption of surfactant molecules at the water–air and solid–water interfaces can significantly change the water surface tension and solid–water interface tension. In turn, these changes influence the contact angle of water on a given solid. In addition, due to the adhesion of surfactant molecules to the solid surface, they can change the morphology and hydrophilic-hydrophobic properties of a solid surface and they can influence the adhesion of other substrates, for example microorganisms [4,5]. This change and influence depend on the solid, surfactant and substrate properties. The antimicrobial properties of surfactants are especially desirable here.

Sucrose fatty acid esters (SE) belong to the non-ionic, non-toxic, non-allergenic, biodegradable and biocompatible sugar-based surfactants obtained by enzymatic or chemical synthesis [6,7,8,9,10,11]. Due to their good surface and aggregation properties, they are used as emulsifiers, solubilizers and stabilizers in various industries. They are applied in pharmacy and medicine and as food additives. Sucrose fatty acid esters are completely safe for the human body and many of them possess antibacterial properties [12,13,14]. Compounds used as food additives and having all the above-mentioned properties simultaneously are not common. Therefore, in recent years the interest in sucrose esters as food additives and permeability enhancers of biologically active substances through the biological membranes has increased [6].

Sucrose fatty acid esters belong to the group of sugar surfactants including for example alkylglucosides, which affect the microorganisms’ adhesion and biofilm formation on the solid substrates [15]. The biofilm is an adherent, matrix-enclosed bacterial population and in nature usually refers to microorganisms’ deposition at the solid–water interface. In general, it is also resistant to antibiotics and physical treatments. Adhesion of bacteria to the surface of biomaterials is an important factor in the pathogenesis of infections; however, the molecular and physical interactions that govern this process are not well understood and explained [5]. The adhesion of microorganisms to a given surface characterized by specific hydrophobic-hydrophilic properties and a specific application depends on both the character of the surface and the substrate undergoing adsorption or adhesion. The bacterial adhesion process is quite complicated and depends on many factors such as the type of bacteria and surface (composition, hydrophobicity and surface roughness), environmental factors, e.g., the presence of protein serum or antibiotics. One of the adhesion of microorganisms to the surface and the biofilm formation inhibition methods is a change in wettability of a given solid by using the surface-active agents (especially biosurfactants, natural surfactants and their mixtures) and change of the hydrophilic-hydrophobic character of a given solid or (bio)material [5]. The adsorption of surfactants can also change the hydrophobicity of the microbial. However, the literature lacks systematic papers studying the influence of antimicrobial sucrose fatty acid esters adsorption on the wettability of solids despite their wide practical application in different areas.

From a practical point of view, it is also very important to study the solid wettability prediction in the systems including sucrose fatty acid esters or other sugar-based surfactants and solids with different polarity as well as those applied in different areas of medicine. Our previous studies on the wettability of apolar polymers by aqueous solutions of surfactants [16,17] report that it is possible to predict the wettability process (contact angle value) in the systems including apolar polymers whose surface tension results only from the Lifshitz-van der Waals intermolecular interactions based on the Lifshitz-van der Waals component of water surface tension as well as aqueous solution of surfactant and solid surface tension.

**Scheme 1:**
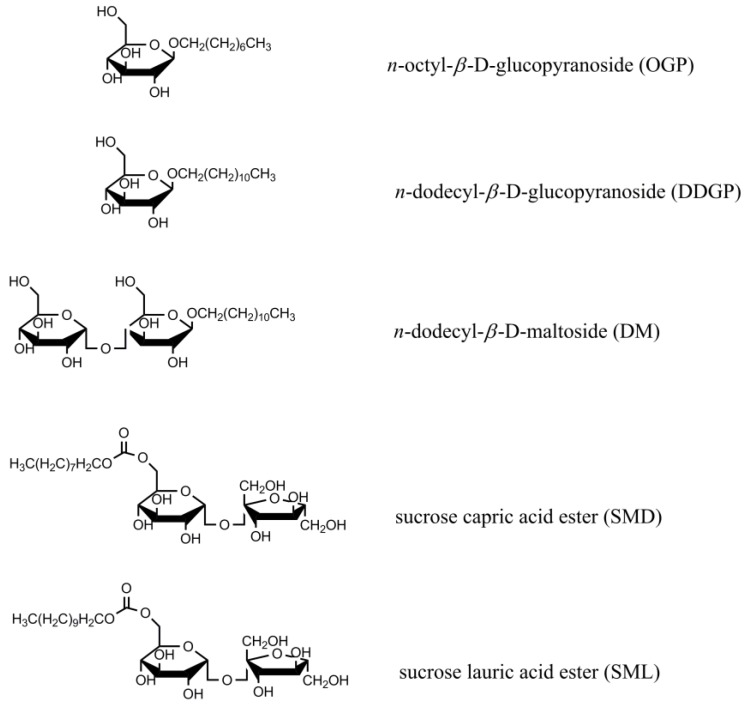
The molecular structure of sugar-based surfactants.

Thus, the purpose of this study was to determine the relationship between the adsorption, adhesion and wetting properties of sucrose capric acid ester (sucrose monodecanoate) (SMD) and sucrose lauric acid ester (sucrose monolaurate) (SML) in the solid-aqueous solution of surfactant-air systems. It is well known that the structure of polar and apolar parts of studied surfactants influences largely on the antimicrobial and adhesion properties of surfactants. Thus, the same considerations were made for the systems including alkylglucoside (*n*-octyl-*β*-d-glucopyranoside (OGP) and *n*-dodecyl-*β*-d-glucopyranoside (DDGP)) and alkylpolyglucoside (*n*-dodecyl-*β*-d-maltoside (DM)) surfactants. The structure of studied surfactants is presented in Scheme 1. The other objective of the paper was to study the wettability prediction in the systems including sucrose fatty acid esters and some other sugar-based surfactants. The studied solids (polyterafluoroethylene (PTFE), polyethylene (PE), poly(methyl methacrylate) (PMMA), polyamide (nylon 6) and quartz) were chosen because of their wide practical applications as well as different polarity.

## 2. Results and Discussion

### 2.1. Contact Angle Values Changes Due to the Influence of Sugar-Based Surfactants

**Figure 1:**
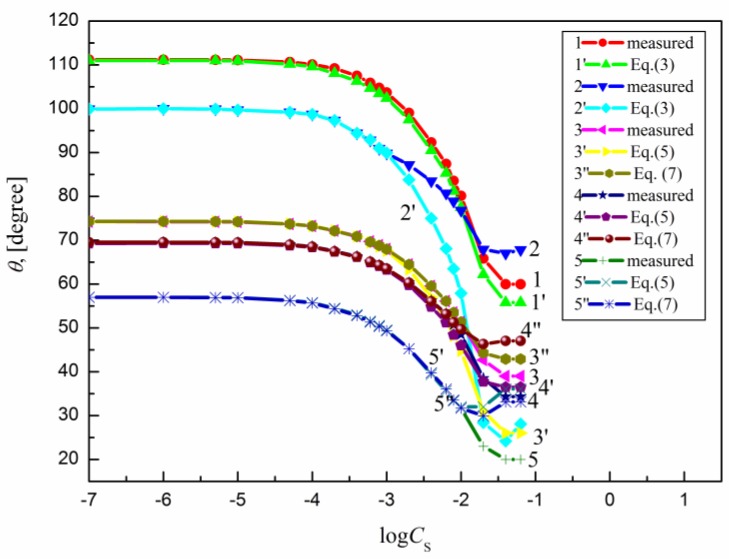
A plot of the contact angle (θ) of the aqueous solution of OGP on the PTFE (curves 1 and 1′), PE (curves 2 and 2′), PMMA (curves 3, 3′ and 3′′), nylon 6 (curves 4, 4′ and 4′′) and quartz (curves 5, 5′ and 5′′) against the logarithm of OGP concentration ($C_{S}$). Curves 1–5—the measured θ values, curves 1′ and 2′—the θ values calculated from Equation (3), curves 3′, 4′ and 5′—the θ values calculated from Equation (5) and curves 3′′, 4′′ and 5′′—the θ values calculated from Equation (7).

**Figure 2:**
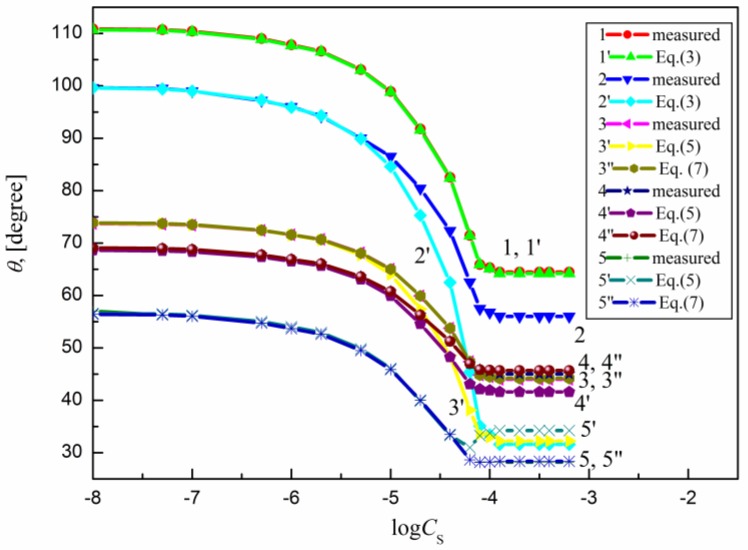
A plot of the contact angle (θ) of the aqueous solution of DDGP on the PTFE (curves 1 and 1′), PE (curves 2 and 2′), PMMA (curves 3, 3′ and 3′′), nylon 6 (curves 4, 4′ and 4′′) and quartz (curves 5, 5′ and 5′′) against the logarithm of DDGP concentration ($C_{S}$). Curves 1–5—the measured θ values, curves 1′ and 2′—the θ values calculated from Equation (3), curves 3′, 4′ and 5′—the θ values calculated from Equation (5) and curves 3′′, 4′′ and 5′′—the θ values calculated from Equation (7).

**Figure 3:**
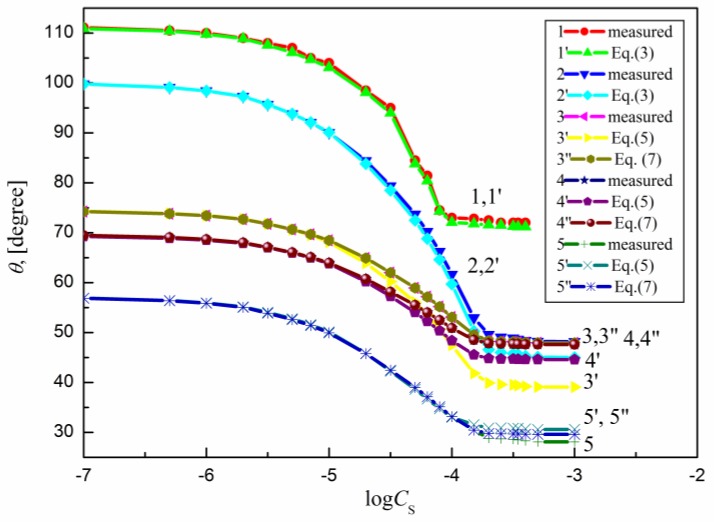
A plot of the contact angle (θ) of the aqueous solution of DM on the PTFE (curves 1 and 1′), PE (curves 2 and 2′), PMMA (curves 3, 3′ and 3′′), nylon 6 (curves 4, 4′ and 4′′) and quartz (curves 5, 5′ and 5′′) against the logarithm of DM concentration ($C_{S}$). Curves 1–5—the measured θ values, curves 1′ and 2′—the θ values calculated from Equation (3), curves 3′, 4′ and 5′—the θ values calculated from Equation (5) and curves 3′′, 4′′ and 5′′—the θ values calculated from Equation (7).

**Figure 4:**
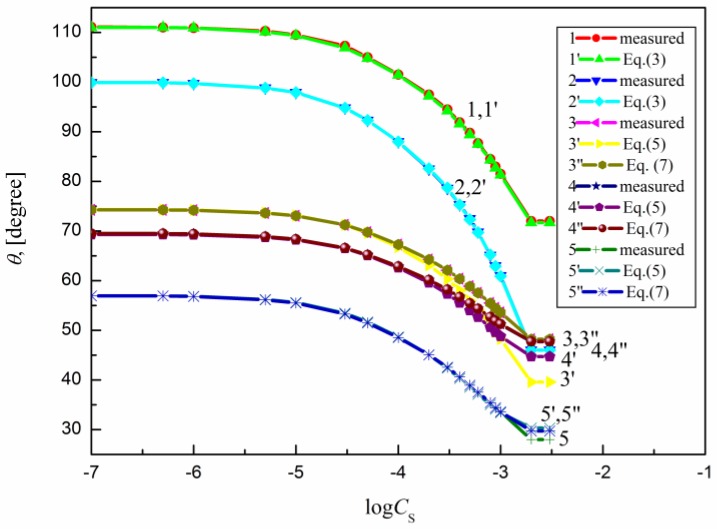
A plot of the contact angle (θ) of the aqueous solution of SMD on the PTFE (curves 1 and 1′), PE (curves 2 and 2′), PMMA (curves 3, 3′ and 3′′), nylon 6 (curves 4, 4′ and 4′′) and quartz (curves 5, 5′ and 5′′) against the logarithm of SMD concentration ($C_{S}$). Curves 1–5—the measured θ values, curves 1′ and 2′—the θ values calculated from Equation (3), curves 3′, 4′ and 5′—the values calculated from Equation (5) and curves 3′′, 4′′ and 5′′—the θ values calculated from Equation (7).

**Figure 5:**
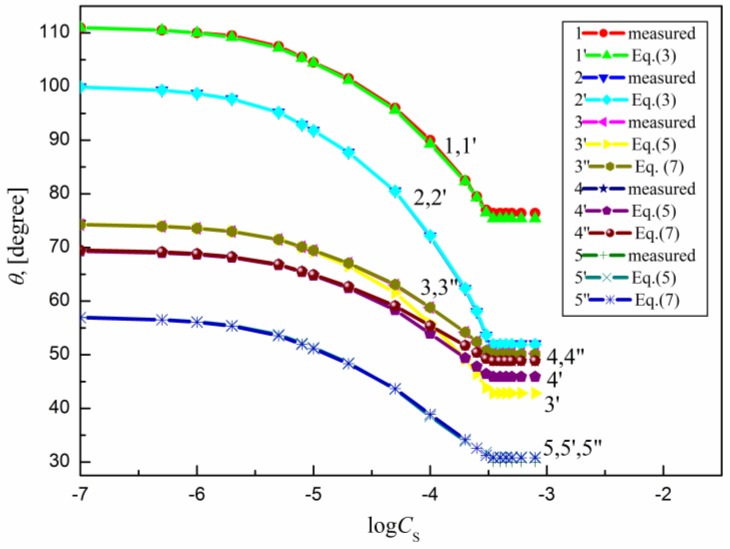
A plot of the contact angle (θ) of the aqueous solution of SML on the PTFE (curves 1 and 1′), PE (curves 2 and 2′), PMMA (curves 3, 3′ and 3′′), nylon 6 (curves 4, 4′ and 4′′) and quartz (curves 5, 5′ and 5′′) against the logarithm of SML concentration ($C_{S}$). Curves 1–5—the measured θ values, curves 1′ and 2′—the θ values calculated from Equation (3), curves 3′, 4′ and 5′—the θ values calculated from Equation (5) and curves 3′′, 4′′ and 5′′—the θ values calculated from Equation (7).

The measured contact angle (θ) values for the aqueous solutions of sucrose lauric acid ester (SML), sucrose capric acid ester (SMD), *n*-octyl-*β*-d-glucopyranoside (OGP), *n*-dodecyl-*β*-d-glucopyranoside (DDGP) and *n*-dodecyl-*β*-d-maltoside (DM) on the PTFE, PE, PMMA, nylon 6 and quartz surface are presented in Figure 1, Figure 2, Figure 3, Figure 4 and Figure 5. For these solids, considering wettability, the literature data of the aqueous solutions of OGP on the PTFE and PE surface [16,18] were taken into account and are presented in Figure 1, Figure 2, Figure 3, Figure 4 and Figure 5 for comparison. From the above-mentioned contact angle values on the PTFE, PE, PMMA, nylon 6 and quartz surface it can be seen that the wettability of a given solid depends on the type (molecular structure) (Scheme S1) of surfactant and its concentration. In every case the contact angle isotherms (Figure 1, Figure 2, Figure 3, Figure 4 and Figure 5) show a characteristic inflection point behind which the contact angle values are almost constant. For a given surfactant and all studied solids the surfactant concentration corresponding to that region is close to that of its critical micelle concentration (CMC) determined earlier from the surface tension and some other measurements [19,20,21,22,23,24,25,26,27,28,29,30]. The values of both CMC and the contact angle at CMC ($\theta_{CMC}$) obtained from the contact angle isotherms for particular surfactants are presented in Table 1. From this table it can be seen that in the case of alkylglucopranoside surfactants (especially OGP) there are some discrepancies between the CMC values determined from the isotherms of contact angle on PE and other solids. This is probably connected with the changes of the PE surface tension because of migration of surfactant molecules on the PE surface and the surfactant film formation around the solution drop settled on the PE. The presence of the film causes the reduction of the PE surface tension. The greater this reduction is, the shorter the hydrophobic chain is in the surfactant tail. The presence of the surfactant film around the drop settled on the PE should also influence its adsorption at the solid–water interface which will be discussed in the coming paragraphs.

From Figure 1, Figure 2, Figure 3, Figure 4 and Figure 5 it can be seen that there is no complete wetting in any case of the studied solids (also for quartz) by surfactant aqueous solutions even at the surfactant concentration higher than its CMC. The minimal contact angle values on a given solid depend on both the length of sugar-based surfactant tail and type of its polar part (glucose, maltose or sucrose) (Figure 1, Figure 2, Figure 3, Figure 4 and Figure 5). On the other hand, the minimal θ values of aqueous solutions of studied surfactants on a given solid are close to those of classical anionic and cationic surfactants but they are somewhat larger than those of classical nonionic ones [31]. From Figure 1, Figure 2, Figure 3, Figure 4 and Figure 5 and the literature data [29,30] it can be seen that the contact angle isotherms of aqueous solutions of studied surfactants for PTFE and PE are similar to those of the studied surfactants surface tension. This suggests that the properties of the adsorption monolayer at the water–air and apolar polymer–water interface are also similar.

It is commonly known that the shape of the contact angle isotherms depends on the solution ($\gamma_{LV}$) and solid ($\gamma_{SV}$) surface tension as well as the solid–solution interface tension ($\gamma_{SL}$). The relationship between these parameters can be described by the Young equation which has the following form [1]:

(1) $$\gamma_{SV}-\gamma_{SL}=\gamma_{LV}\cos\theta$$

From Equation (1) it can be seen that if $\gamma_{SV}$ is constant during the wettability of a given solid by the aqueous solutions of studied surfactant in the studied concentration range, then the θ values depend on the $\gamma_{LV}$ and $\gamma_{SL}$ ones. Taking this into account it was interesting to examine whether it is possible to predict (calculate) the contact angle values of aqueous solutions of SMD, SML, OGP, DDGP and DM on PTFE, PE, PMMA, nylon 6 and quartz surface.

For this purpose, the following equation can be used [32,33,34,35]:

(2) $$\gamma_{LV}\left(\cos\theta +1\right)=2\left(\sqrt{\gamma_{L}^{LW}\gamma_{S}^{LW}}+\sqrt{\gamma_{L}^{+}\gamma_{S}^{-}}+\sqrt{\gamma_{L}^{-}\gamma_{S}^{+}}\right)$$

where $\gamma_{L}^{LW}$ and $\gamma_{S}^{LW}$ are the Lifshitz-van der Waals component of the solution and solid surface tension, $\gamma_{L}^{+}$ and $\gamma_{S}^{+}$ are the electron-acceptor parameter of the acid-base component of solution ($\gamma_{L}^{AB}$) and solid ($\gamma_{S}^{AB}$) surface tension, $\gamma_{L}^{-}$ and $\gamma_{S}^{-}$ are the electron-donor parameters of the acid-base component of solution and solid surface tension, respectively.

In the case of solids whose surface tension results only from the Lifshitz-van der Waals intermolecular interactions Equation (2) is as follows [32]:

(3) $$\gamma_{LV}\left(\cos\theta +1\right)=2\sqrt{\gamma_{L}^{LW}\gamma_{S}^{LW}}$$

For these calculations the values of solid surface tension ($\gamma_{SV}$) as well as its components and parameters obtained earlier [36,37,38] should be considered (Table 2). The $\gamma_{SV}$ of PTFE was determined earlier based on the contact angle of *n*-alkanes and is equal to 20.24 mN/m [37]. For the calculations of θ the components and parameters of the solution surface tension must be also known. Based on the results obtained earlier [39] it was assumed that the decrease of the water surface tension under the influence of surfactants is practically associated with the decrease of acid-base component ($\gamma^{AB}$) of this tension and that the electron-acceptor and electron-donor parameters are equal to those for pure water (Table 2).

The Lifshitz-van der Waals ($\gamma_{}^{LW}$) component of the solution surface tension equal to that of water (26.85 mN/m) [38] was also used for the contact angle calculations.

Because PTFE and PE belong to the apolar hydrophobic polymers Equation (3) was used for the calculations of the contact angle on these solids. The contact angle values on the PTFE, PE, PMMA, nylon 6 and quartz calculated in such a way are presented in Figure 1, Figure 2, Figure 3, Figure 4 and Figure 5. From these figures it can be seen that there is a good agreement between the measured and calculated θ values of aqueous solutions of all studied surfactants on the PTFE surface as well as in the case of the aqueous solutions of sucrose esters on the PE surface. Accordingly, the properties of the sucrose esters adsorption layers at the water–air and low-energetic polymer–water interfaces are the same in the whole surfactant concentration range or the packing and orientation of SE molecules at the interfaces are similar. This can be stated for OGP, DDGP and DM only in the case of PTFE.

This also proves the usefulness of the new Lifshitz-van der Waals component of the water surface tension in the contact angle prediction in the systems including some apolar polymers. In the case of OGP and DDGP (monosaccharide-based surfactants) there is a good agreement between the measured and calculated from Equation (3) contact angle values on PE but only in the range of surfactant concentration corresponding to the unsaturated monolayer at the water–air interface [29,30]. For DM much smaller differences were observed in the measured and calculated θ on PE. In the case of PE these discrepancies probably result from the fact that PE surface tension changes due to the surfactant film formation around the solution drop. As these changes depend mainly on the length of the surfactant tail (its surface tension) (Table 2), the greatest differences between the measured and calculated contact angle values are in the case of OGP.

If during the wettability process $\gamma_{SV}$ of a given solid is changed because of the penetration of surfactant molecules on the solid surface, then Equation (1) should be written as follows:

(4) $$\gamma_{S}-\pi_{e}-\gamma_{SL}=\gamma_{LV}\cos\theta$$

and Equation (2) should be presented as:

(5) $$\gamma_{LV}\left(\cos\theta +1)\right)=2\left(\sqrt{\gamma_{L}^{LW}\gamma_{S}^{LW}}+\sqrt{\gamma_{L}^{+}\gamma_{S}^{-}}+\sqrt{\gamma_{L}^{-}\gamma_{S}^{+}}\right)-\pi_{e}$$

where $\pi_{e}$ is the surfactant film pressure.

To test whether the surfactant layer influences on the surface tension of a solid the Neumann et al. equation was applied [40,41]:

(6) $$\frac{\cos\theta +1}{2}=\sqrt{\frac{\gamma_{S}}{\gamma_{L}}}\exp\left[-\beta {\left(\gamma_{L}-\gamma_{S}\right)}^{2}\right]$$

where according to Neumann et al. β is the constant for all systems and its most proper value is equal to 0.000115 (m^2^/mJ)^2^.

It occurs that for all studied systems including sugar-based surfactants as well as polar polymers and quartz the values of solid surface tension calculated from Equation (6) are changed as a function of surfactants concentration. This proves that in such a case the surfactant layer is probably formed around the drop settled on the solid and changes this tension. For these reasons, Equation (5) instead of Equation (2) was used here for calculations of θ on the PMMA, nylon 6 quartz surface. The $\pi_{e}$ values in this equation calculated from Equation (6) are equal to the difference between the solid surface tension calculated for pure water and that calculated for aqueous solution of surfactant at a given concentration. The θ values calculated in such a way are presented in Figure 1, Figure 2, Figure 3, Figure 4 and Figure 5. These results show that there is a good agreement between the measured and calculated from Equation (5) θ values especially in the surfactant concentration range corresponding to the unsaturated surfactant monolayer at the water–air interface. In the case of quartz there is a good agreement between the measured contact angle values and those calculated from Equation (5) (Figure 1, Figure 2, Figure 3, Figure 4 and Figure 5) practically up to the surfactant concentration just above its CMC. The best agreement was found in the case of DM and sucrose esters. This suggests that DM and sucrose esters molecules orientation toward the quartz–water interface practically does not depend on the surfactant concentration.

In general, the greatest differences between the values of the measured and calculated from Equation (5) contact angle on PMMA, nylon 6 and quartz occur in the case of OGP. It probably results from the fact that the solid surface tension can be reduced by the presence of the sugar surfactant film around the drop settled on the solid surface. This reduction depends on the orientation of surfactant molecules toward the solid–water interface and solid as well as head and tail of surfactant surface tension. In the case of the water–air interface the water surface tension changes from the water surface tension to the surfactant tail surface tension. In the case of the polar solids–water interface where the surfactant molecule is parallel oriented the solid surface tension changes to the average value of the tail and head surface tension. Thus, at the first approximation it was assumed that the maximal difference between the solid surface tension and the surface tension of the solid with the surfactant film is equal to $\pi_{e}$/2. In such a case Equation (5) should be as follows:

(7) $$\gamma_{LV}\left(\cos\theta +1)\right)=2\left(\sqrt{\gamma_{L}^{LW}\gamma_{S}^{LW}}+\sqrt{\gamma_{L}^{+}\gamma_{S}^{-}}+\sqrt{\gamma_{L}^{-}\gamma_{S}^{+}}\right)-\frac{\pi_{e}}{2}$$

Surprisingly the values of measured contact angle on PMMA, nylon 6 and quartz surface are practically fully compatible with those calculated from Equation (7) (Figure 1, Figure 2, Figure 3, Figure 4 and Figure 5). However, still the largest differences occur in the case of OGP. This probably results from the fact that in the case of OGP the reduction of the solid surface tension is the largest due to the shortest alkyl chain in its hydrophobic part.

The changes of the contact angle values should be also reflected in the solid–solution interface tension changes.

### 2.2. Solid–Liquid Interface Tension Changes Due to the Influence of Sugar Surfactants

Knowing the surface tension of studied solids (Table 2) and the contact angle values of aqueous solutions of studied surfactants the solid–water interface tension was calculated from Equation (1) or Equation (4). The obtained values of $\gamma_{SL}$ are presented in Figures S1–S5 from which it can be seen that in the case of all studied solids and surfactants their addition to water causes the $\gamma_{SL}$ changes. These figures show that $\gamma_{SL}$ decreases with the increasing surfactant concentration which indicates that surfactant adsorption rises and causes these changes.

**Figure 6:**
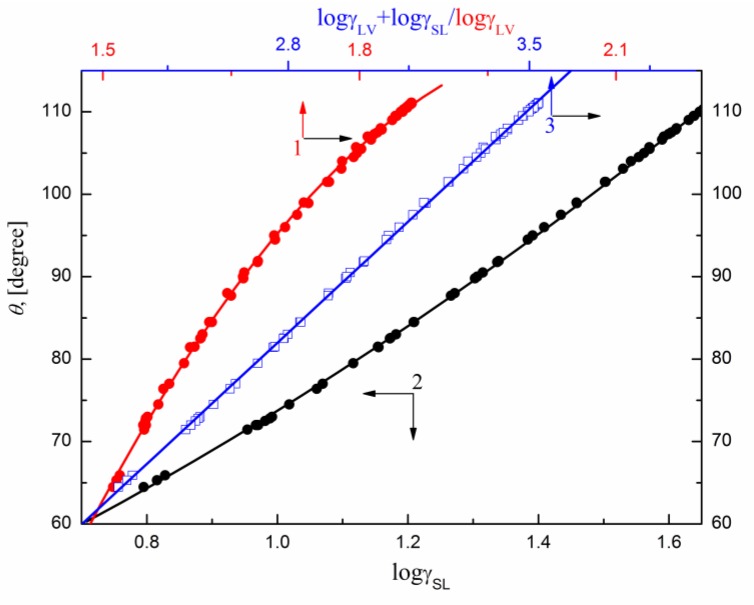
A plot of the contact angle (θ) of the aqueous solution of DDGP, DM, SMD and SML on the PTFE surface vs. the surface tension of solution ($\gamma_{LV}$) (curve 1), PTFE–solution interface tension ($\gamma_{SL}$) (curve 2) and $\log_{LV}+\log_{SL}$ (curve 3).

It appeared that in the case of PTFE the relationships between θ and the sum of logarithms of surface tension and solid–water interface tension can be described by one linear function (Figure 6) and those between θ vs. $\log\gamma_{LV}$ and θ vs. $\log\gamma_{SL}$ by the second order polynomial one. This indicates that in the case of PTFE the $\gamma_{LV}$ and $\gamma_{SL}$ influence θ values to the same extent and that $\gamma_{SV}$ of PTFE does not change during the wettability process. This also means that the orientation of the studied surfactant molecules toward the water–air and PTFE–water interface is similar.

**Figure 7:**
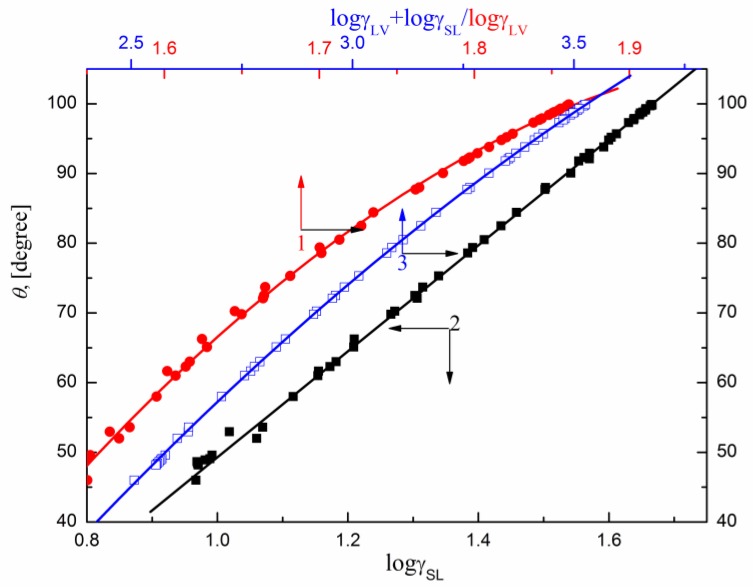
A plot of the contact angle (θ) of the aqueous solution of SMD and SML on the PE surface vs. the surface tension of solution ($\gamma_{LV}$) (curve 1), PTFE–solution interface tension ($\gamma_{SL}$) (curve 2) and $\log_{LV}+\log_{SL}$ (curve 3).

Contrary to PTFE, in the case of PE (Figure 7) the relationship between θ vs. $\log\gamma_{SL}$ is linear in the case of all studied surfactants but only in the case of sucrose esters it can be described by one linear function. This is probably connected with the fact that in the case of DDGP and DM $\gamma_{SV}$ of PE changes because of the penetration of surfactant molecules on the solid surface and the surfactant film is formed around the solution drop settled on the PE surface. This may also result (similarly to OGP) from the changes in the surface tension of the solution being in contact with the PE surface [16]. In the case of sucrose esters, the orientation of their molecules toward the PE–water and water–air interface is also similar.

The discrepancies between the measured and calculated contact angle values on a given solid can result from different orientation of surfactant molecules toward the water–air and solid–water interfaces. Three cases of surfactant molecules orientation at the solid–water interface are possible: (a) perpendicular orientation by the hydrophilic (sugar) head toward the air phase, (b) perpendicular orientation by the hydrophobic tail toward the air phase and (c) parallel orientation of surfactant molecules.

If we assume that the surfactant molecules are oriented in the surface layer by the hydrophilic head toward the air phase, then the surface tension of the surfactants should be close to that of surfactant head (Table 2). Thus, in such a case the contact angle values can be calculated from Equation (2) based on the solid and surfactant head surface tension as well as their components and parameters (Table 2).

If the surfactant molecules are oriented by the hydrophobic tail toward the air phase, then the surface tension of the surfactants should be close to that of surfactant tail (Table 2). In such a case the contact angle of aqueous solution on a given solid can be calculated from Equation (3) in which $\gamma_{S}^{LW}$ is equal to the tail surface tension of particular surfactants (Table 2) [42]. If the surfactant molecules are assumed to be parallel oriented toward the solid–air interface, the contact angle values depend on tail and head of surfactants surface tension and their contactable area. For such a case the Baxter and Cassie equation should be applied for contact angle calculation [43,44]:

(8) $$\cos\theta =x_{1}\cos\theta_{1}+x_{2}\cos\theta_{2}$$

where $\theta_{1}$ is the contact angle of solution on the surfactant tail, $\theta_{2}$ is the contact angle of solution on the surfactant head, $x_{1}$ and $x_{2}$ are the fractions of surface occupied by the tail and head of surfactants, respectively.

The contactable area of tail and head of particular surfactants (used for the fraction of area occupied by the tail and head of surfactant at the solid–water interface calculations) was determined earlier from the length of bonds and the angle between them [45].

**Figure 8:**
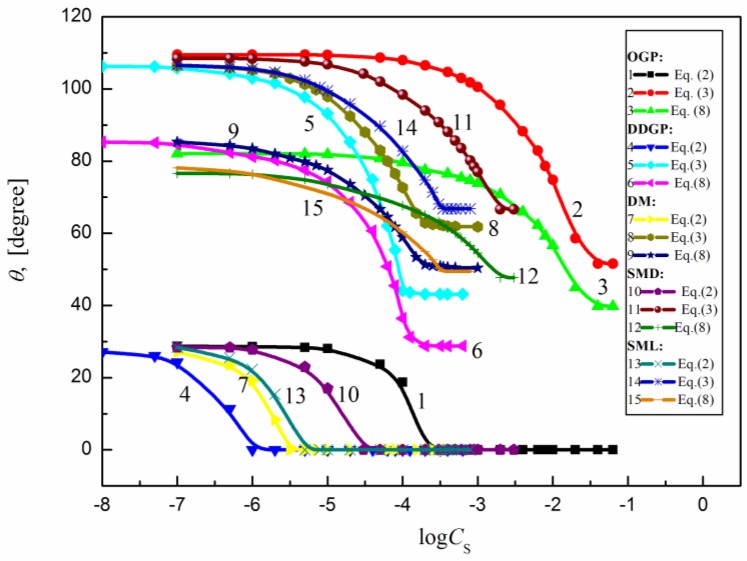
A plot of the contact angle (θ) of the aqueous solution of OGP (curves 1–3), DDGP.(curves 4–6), DM (curves 7–9), SMD (curves 10–12) and SML (curves 13–15) calculated from Equation (2) (perpendicular orientation by the hydrophilic head toward the air phase), Equation (3) (perpendicular orientation by the hydrophobic tail of surfactant toward the air phase) or Equation (7) (parallel orientation of surfactant molecules toward the interface region).

The calculated contact angle values for particular surfactants on the assumption of proper orientation of their molecules are presented in Figure 8.

**Scheme 2:**
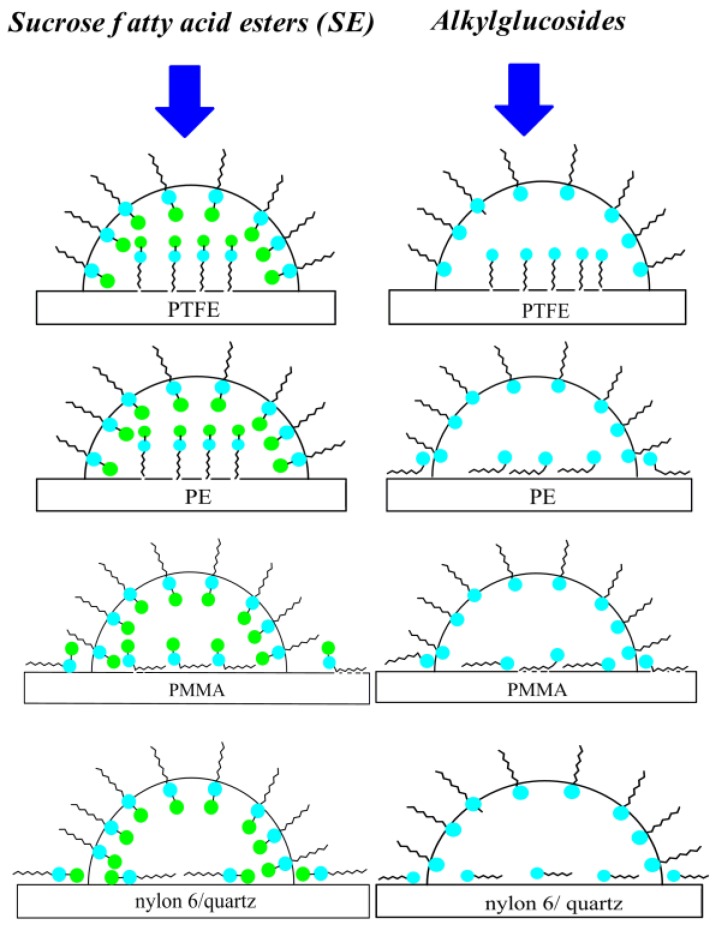
The orientation of studied surfactant molecules toward the solid–water interface.

Because of good agreement between the calculated and measured contact angle values on PTFE (in the case of all studied surfactants) and PE (in the case of sucrose esters) it can be stated that the orientation of surfactant molecules toward the PTFE/PE–water and water–air interface is perpendicular [29,30] (Scheme 2).

As can be seen from Figure 8 and Scheme 2 in the case of PMMA, nylon 6 and quartz the studied surfactant molecules orientation toward the interface at the saturated state is rather parallel because the minimal measured contact angle values are close to those calculated from Equation (8). In addition, because the Lifshitz-van der Waals component of the surfactant head surface tension is close to that of the PMMA, nylon 6 and quartz surface it can be stated that the interactions between the head of surfactant and the solid surface are stronger that those between the tail and solid.

In the case of quartz, the minimal contact angle values are lower than those calculated from Equation (8) which can be associated with a small surface coverage by surfactant molecules. Moreover, it is commonly known that the vapor film behind the settled drop can influence the contact angle value but the role of vapor film cannot be taken into account in any equation used for the components and parameters of the quartz surface tension determination. The surfactant molecule orientation at the solid–water interface also affects its concentration at the interface.

### 2.3. Concentration of Sugar–Based Surfactants at the Solid–Water Interface

A relative amount of adsorbed surfactant at the solid–water interface can be obtained from the Lucassen-Reynders equation [1,46] which has the form:

(9) $$\frac{d\left(\gamma_{LV}\cos\theta \right)}{d\gamma_{LV}}=\frac{\Gamma_{SV}-\Gamma_{SL}}{\Gamma_{LV}}$$

where $\Gamma_{SL}$, $\Gamma_{LV}$ and $\Gamma_{SV}$ is the surface excess concentration of surfactant at the solid–water, water–air and solid–air interfaces, respectively and $\gamma_{LV}\cos\theta$ is the adhesion tension. Accordingly, it is possible to determine the $\frac{\left(\Gamma_{SV}-\Gamma_{SL}\right)}{\Gamma_{LV}}$ ratio. If $\Gamma_{SV}$ is constant in the total range of surfactant concentration (*C_S_*), it is possible to determine the $\Gamma_{SL}$ knowing the $\Gamma_{LV}$. It appeared that in the case of PTFE and for the aqueous solutions of all studied surfactants there is a linear dependence between the adhesion and surface tension (Figure S6) which can be described by one equation:

(10) $$\gamma_{LV}\cos\theta =-1.0013\gamma_{LV}(\pm 4.1964\times 10^{-4})+46.5636(\pm 0.0217)$$

In the case of PE there is a linear dependence between the adhesion and surface tension (Figure S7) only for sucrose esters which can be described by one equation:

(11) $$\gamma_{LV}\cos\theta =-0.9999\gamma_{LV}(\pm 6.8067\times 10^{-4})+60.1688(\pm 0.0362)$$

On the other hand, the relationship between $\gamma_{LV}\cos\theta$ and $\gamma_{LV}$ for OGP [18], DDGP and DM can be divided into two parts. The inflection point exists at the OGP, DDGP and DM concentration referring to their saturated monolayer formation at the water–air interface [29,30]. It is interesting that in the low surfactant concentration region (before the inflection point) the equation describing the $\gamma_{LV}\cos\theta$ vs. $\gamma_{LV}$ relationship for both surfactants is practically the same as for sucrose esters but behind that point it is somewhat different for each alkylglucoside-based surfactant. For DDGP behind the inflection point this relationship can be described by the following equation:

(12) $$\gamma_{LV}\cos\theta =-0.6586\gamma_{LV}(\pm 5.7384\times 10^{-5})+39.5788(\pm 0.0022)$$

and for DM:

(13) $$\gamma_{LV}\cos\theta =-0.9567\gamma_{LV}(\pm 0.0018)+57.2474(\pm 0.0940)$$

In the case of PMMA, nylon 6 and quartz the $\gamma_{LV}\cos\theta$ vs. $\gamma_{LV}$ relationship cannot be described by the linear expression independently of the surfactant concentration range (Figures S7–S10). However, in the case of PMMA or quartz this relationship is practically the same for all studied surfactants. In addition, in the case of PMMA (Figure S8) in the surfactant concentration range corresponding to the unsaturated monolayer at the water–air interface this relationship can be described by one linear equation for most studied surfactants:

(14) $$\gamma_{LV}\cos\theta =-0.1848\gamma_{LV}(\pm 0.0011)+33.1820(\pm 0.0761)$$

Based on the obtained results (Figures S6–S10) and assuming that $\Gamma_{SV}$ is constant, it can be stated that the adsorption of all studied surfactants at the water–air and PTFE–water interfaces is practically the same. Adsorption of sucrose esters at the PE–water and water–air interface is also the same.

On the other hand, Equation (9) does not provide any information about the surface excess concentration of the surface-active agents at the solid–solution ($\Gamma_{SL}$) and solid–air interfaces.

$\Gamma_{SL}$ can be directly determined from the Gibbs isotherm adsorption equation knowing the changes of $\gamma_{SL}$ as a function of surfactant concentration using the following equation [1]:

(15) $$\Gamma_{SL}^{}=-\frac{C_{S}}{nRT}{\left(\frac{\partial \gamma_{SL}}{\partial C_{S}}\right)}_{T}=-\frac{1}{nRT}{\left(\frac{\partial \gamma_{SL}}{\partial \ln C_{S}}\right)}_{T}=-\frac{1}{2.303nRT}{\left(\frac{\partial \gamma_{SL}}{\partial \log C_{S}}\right)}_{T}$$

where *n* is the number depending on the kind of surfactant which was assumed to be equal to 1 for nonionic ones, R is the gas constant and T is the temperature.

**Figure 9:**
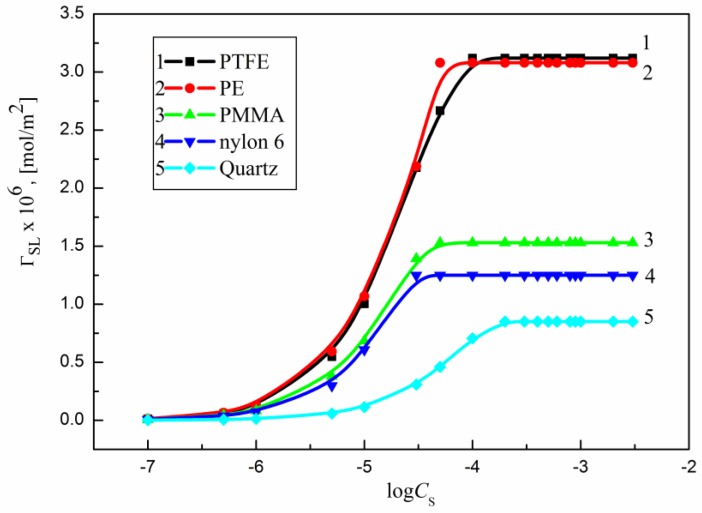
A plot of the SMD Gibbs surface excess concentration ($\Gamma_{SL}$) (curves 1–5) at the PTFE, PE, PMMA, nylon 6 or quartz–water interfaces, respectively, vs. the logarithm of SMD concentration ($\log C_{S}$).

**Figure 10:**
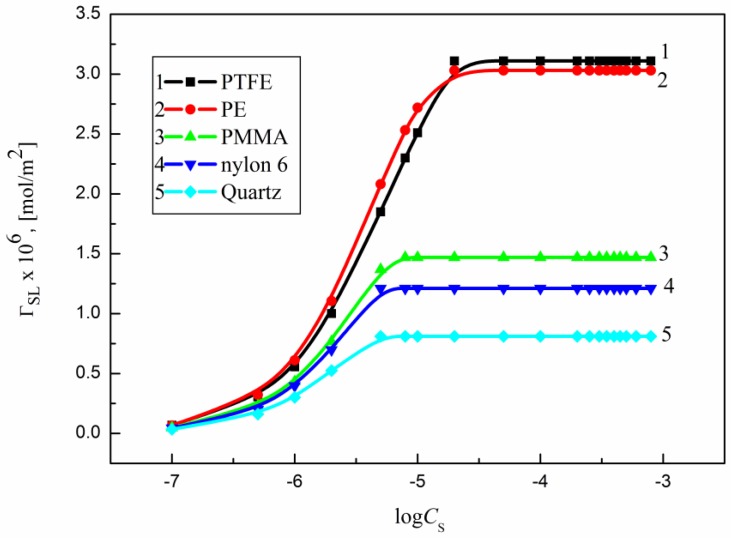
A plot of the SML Gibbs surface excess concentration ($\Gamma_{SL}$) (curves 1–5) at the PTFE, PE, PMMA, nylon 6 or quartz–water interfaces, respectively, vs. the logarithm of SML concentration ($\log C_{S}$).

Assuming that $\Gamma_{SV}$ is constant, it was possible to calculate the PTFE–water interface tension for all studied surfactants and the PE–water one for sucrose esters from Equation (1) (Figures S1 and S2). In the case of PMMA, nylon 6 and quartz, the solid–water interface tension was calculated from Equation (4) (Figures S3–S5) where solid surface tension changes were considered and determined from Equation (6). For DDGP and DM the PE–water interface tension was calculated from both Equations (1) and (4). Next, the relationship between the $\gamma_{SL}$ and surfactant concentration was established. In the surfactant concentration range from 0 to that corresponding to the saturated monolayer it was possible to describe this relationship by the second order exponential function. Based on Equation (15) the $\Gamma_{SL}$ values for all studied systems were determined and are presented in Figure 9, Figure 10 and Figures S11–S13.

The maximal $\Gamma_{SL}^{}$ values ($\Gamma_{SL}^{\max}$) corresponding to the saturated adsorption monolayer of surfactant at the solid–water interface (Table 3) were determined from the linear dependence of $\gamma_{SL}$ vs. $\log C_{}$ in every case. $\Gamma_{SL}^{\max}$ or the minimal area ($A_{SL}^{\min}$) (Table 3) [1] of surfactant molecule at the solid–water interface reflects the orientation of surfactant molecules at the solid–water interface in relationship to that at the water–air one.

From these calculations it can be seen that in every case adsorption of surfactant at the PTFE–water interface is practically the same as that at the water–air one. The same adsorption and probably the same orientation of surfactant molecules also take place in the PE-solution-air systems. Moreover, it is found that the $\Gamma_{SL}^{\max}$ values for OGP [18] and DDGP in the case of PTFE and PE are the highest. This results from the fact that these surfactants are more hydrophobic than the others studied, and the interactions between OGP and DDGP molecules and apolar polymer surface are the strongest. For the disaccharide based ones the adsorption of their molecules at a given polymer–water interface is comparable. Thus, the structure and interactions between the surfactant sugar parts (similar to the water–air interface) [29] are decisive regarding the adsorbed surfactant amount at the polymer–water interface.

In the case of PMMA, nylon 6 and quartz in general the $\Gamma_{SL}^{\max}$ values for all studied surfactants are smaller than those at the water–air interface. This points out from the fact that orientation of sugar surfactant molecules at the PMMA/nylon 6/quartz–water interfaces is different from that at the water–air one. In the case of quartz, the $\Gamma_{SL}^{\max}$ values for OGP, DDGP and DM are somewhat higher than those for the disaccharide-based surfactant. In addition, a low adsorption amount of sugar-based surfactants (especially those based on sucrose) at the nylon 6–water and quartz–water interfaces results from the fact that their molecules are oriented parallel toward the interface and in the case of sucrose esters both polar sugar units (glucose and fructose) are located at the solid–water interface.

In the case of PMMA sucrose ester molecules are adsorbed parallel at the solid–water interface by one sugar unit (fructose). The second one (glucose) is directed toward the water phase. This statement can be also proved by the contact angle values (Figure 1, Figure 2, Figure 3, Figure 4 and Figure 5).

The $\Gamma_{SL}^{\max}$ values (Table 3) at the PE–water interface for DDGP and DM determined on the basis of $\gamma_{SL}$ calculated from Equation (1) (Figure S2) and Equation (4) are quite close but still they are not equal to those at the water–air interface. This probably results from the fact that the PE surface tension is changed during the wettability process [16].

### 2.4. Packing of the Surfactant Monolayer at the Solid–Water Interface

From the above-mentioned considerations it can be seen that adsorption of the studied surfactant molecules at the solid–water interface depends mainly on the interactions between the surfactant polar parts. If so, the packing of the adsorbed monolayer at the water–air interface should also reflect them.

The extent of coverage at the solid–air interface by the surfactant molecules can be determined from the following relationship [29,30]:

(16) $$X_{SL}=\frac{\Gamma_{SL}}{\Gamma_{SL}^{\infty }}$$

where $X_{SL}^{}$ is the mole fraction of the area occupied by the molecules of a given surfactant in the adsorption layer and $\Gamma_{SL}^{\infty }$ is the limiting Gibbs surface excess concentration of surfactant at the solid–water interface.

For $\Gamma_{SL}^{\infty }$ the $A_{SL}^{0}$ must be known. Among others, the $A_{SL}^{0}$ values can be determined from the Joos equation of state which for the aqueous solutions of surfactants can be written in the form [47]:

(17) $$\exp\left(\frac{-\pi }{RT\Gamma_{W}^{\infty }}\right)+\exp\left(\frac{-\pi }{RT\Gamma_{SL}^{\infty }}\right)\frac{C_{S}}{a_{SL}^{s}}=1$$

where $\Gamma_{W}^{\infty }$ is the limiting Gibbs surface excess concentration of water at the solid–water interface, π is the film pressure and $a_{S}^{s}$ is the activity of a given surfactant at the solid–water interface. The obtained $\Gamma_{SL}^{\infty }$ and $A_{SL}^{0}$ values for particular surfactants on the studied solids are presented in Table 3.

It should be also remembered that the number of water molecules which can be replaced by one surfactant molecule at the solid–water interface can be equal to the ratio of $\frac{\Gamma_{SL}^{\infty }}{\Gamma_{W}^{\infty }}$. Assuming that $\frac{\Gamma_{SL}^{\infty }}{\Gamma_{W}^{\infty }}=\frac{1}{k}$ [30,39] Equation (17) is as follows:

(18) $$X_{SL}=\frac{1}{k}\frac{\Gamma_{SL}}{\Gamma_{W}+\Gamma_{SL}}$$

The values of $X_{SL}$ for the glucoside and disaccharide-based surfactants calculated from Equation (18), independently of the solid type, are practically the same as those obtained from the $\frac{\Gamma_{SL}}{\Gamma_{SL}^{\infty }}$ ratio (Figures S14–S18). From the obtained $X_{SL}$ values calculated from both Equations (16) and (18) it can be seen that in the case of the disaccharide-based surfactants (SMD, SML and DM) the mole fraction of the area occupied by their molecules at the PTFE/PE–water interface is practically the same as that at the water–air one. This means that bringing the low-energetic surface into contact with the surfactant film coated water the orientation or packing of the film adsorbed at the water–air interface does not change. Surprisingly, at the nylon 6–water interface (Figures S14–S18) the packing of SMD, SML and DM is higher than that of monosaccharide and higher than at the PMMA–water interface. Due to the presence of –NH groups on the nylon 6 surface [36,45] and a larger number of hydroxyl groups in the disaccharide surfactant molecules in comparison to the monosaccharide ones, the surface coverage by SMD, SML and DM is larger than that of OGP and DDGP. Also, the $X_{SL}$ for SMD, SML and DM at the PMMA–water interface (Figures S16–S18) on whose surface only –CH_3_ and =CO groups are practically found [36,45] is the evidence for that statement. From the above considerations and the data presented in Table 3 it can also be seen that the orientation of all studied sugar-based surfactant molecules is parallel at both the nylon 6 and PMMA–water interfaces. In the case of SMD, SML and DM their molecules are located at the nylon 6–water interface with two and at the PMMA–water interface with one sugar unit of surfactant polar part. The quartz surface coverage by the studied sugar surfactant molecules is very small in comparison to the other studied surfaces. This is probably because the thin water film can be formed on the quartz surface which is difficult to remove by sugar surfactant molecules during the quartz wettability process.

### 2.5. Critical Surface Tension of Solid Wetting

From a practical point of view, it is interesting to know the surface tension at which the complete wetting of a given solid occurs. This tension is called the critical surface tension of solid wetting ($\gamma_{C}$) [48] and among others, can be estimated from the relationship: $\gamma_{LV}\cos\theta$ vs. $\gamma_{LV}$ (Figures S6–S10) or cosθ vs. $\gamma_{LV}$ (Figures S19–S23). It turns out that in most systems studied in this paper the $\gamma_{LV}\cos\theta$ vs. $\gamma_{LV}$ dependence can be described by the linear function and that of cosθ vs. $\gamma_{LV}$ by the polynomial one of second order. In the case of the PTFE the relationship $\gamma_{LV}\cos\theta$ vs. $\gamma_{LV}$ (Figure S6) can be described by one linear function for all studied surfactants. In the case of PE for DDGP and DM the $\gamma_{LV}\cos\theta$ vs. $\gamma_{LV}$ and cosθ vs. $\gamma_{LV}$ the relationship’s course can be divided into two parts. The first part is in the range of surfactant concentration corresponding to its unsaturated monolayer at the water–air interface and the second one in the surfactant concentration range corresponding to its saturated monolayer at the water–air interface. Thus, the $\gamma_{C}$ of PE for DDGP and DM was determined only from the first part of $\gamma_{LV}\cos\theta$ vs. $\gamma_{LV}$. The obtained $\gamma_{C}$ values for all studied systems are presented in Table 4.

The average $\gamma_{C}$ value for PTFE determined from the $\gamma_{LV}\cos\theta$ vs. $\gamma_{LV}$ is equal to 23.11 mN/m and similar to the classical surfactants [31], is higher than the PTFE surface tension (20.24 mN/m) determined on the basis of the contact angle for *n*-alkanes [37]. However, the $\gamma_{C}$ values for the PTFE determined from the cosθ vs. $\gamma_{LV}$ relationship is lower than 20.24 mN/m. In the case of PE its $\gamma_{C}$ determined both from $\gamma_{LV}\cos\theta$ vs. $\gamma_{LV}$ and cosθ vs. $\gamma_{LV}$ relations is lower than the PE surface tension (33.71 mN/m). In the case of PMMA and nylon 6 (similar to PE) the reliable values of the critical surface tension of solid wetting were obtained only from the first linear part of the $\gamma_{LV}\cos\theta$ vs. $\gamma_{LV}$ dependence. As it was stated earlier it probably results from the surfactant film formation on the solid surface and solid surface tension changes. The average $\gamma_{C}$ value of PMMA and nylon 6 determined in such a way is equal to 28.30 mN/m and 29.39 mN/m, respectively. The obtained values are much lower than PMMA and nylon 6 surface tension. $\gamma_{C}$ for quartz was determined only from the relationship between cosθ and $\gamma_{LV}$ and its average value is equal to 24.42 mN/m. This value similar to that for PMMA and nylon 6, is lower than the solid surface tension and close to the Lifshitz-van der Waals component of water surface tension (26.85 mN/m). From Table 4 and above-mentioned considerations it can be seen that the $\gamma_{C}$ value depends slightly on the sugar surfactant type and is not equal to the solid surface tension in any case. The $\gamma_{C}$ value is equal to that of solid surface tension if $\gamma_{SV}$ is strictly equal to 26.85 mN/m.

### 2.6. Work of Adhesion

The ability of the surfactant molecules to coat a given solid surface can be estimated and predicted based on the work of adhesion ($W_{A}$). Among others, $W_{A}$ can be determined from the following equation [1]:

(19) $$W_{A}=\gamma_{LV}+\gamma_{SV}-\gamma_{SL}$$

If $\gamma_{SV}$ and $\gamma_{SL}$ are not known and the contact angle (θ) of liquid on the solid surface is higher or strictly equal to zero, then the $W_{A}$ can be calculated from Young-Dupré Equation (1):

(20) $$W_{A}=\gamma_{LV}\left(\cos\theta +1\right)$$

Additionally, if components and parameters of the liquid and solid surface tension are known, the $W_{A}$ can be calculated based on Equations (2), (3) or (5) (in the case of apolar solids).

From Equation (20) it can be seen that if the relationship between $\gamma_{LV}\cos\theta$ and $\gamma_{LV}$ is linear and the slope of the linear dependence is equal to −1, then the constant in this equation is equal to $W_{A}$ [49]:

(21) $$\gamma_{LV}\cos\theta =-\gamma_{LV}+W_{a}$$

The $W_{A}$ values for particular studied systems calculated from Equation (20) and from Equations (3) and (5) or (7) are presented in Figures S24–S26.

In the case of PTFE (for all studied surfactants) and PE (for sucrose esters) there is one linear relationship between $\gamma_{LV}\cos\theta$ and $\gamma_{LV}$ (Figures S6 and S7). The average $W_{A}$ value for PTFE estimated from the above mentioned dependence is equal to 46.31 mJ/m^2^ and is very close to that calculated from Equation (3) (46.62 mJ/m^2^) in which the Lifshitz-van der Waals components of water (26.85 mN/m) and PTFE (20.24 mN/m) surface tension were applied. It proved that $W_{A}$ of the aqueous solutions of sugar surfactants to PTFE is similar to that determined earlier for the classical surfactants and biosurfactants [16].

The average $W_{A}$ value for the aqueous solutions of sucrose ester to the PE surface calculated on the basis of $\gamma_{LV}\cos\theta$ vs. $\gamma_{LV}$ relationship (60.17 mJ/m^2^) is equal to that calculated from Equation (3). In the case of glucose-based surfactants (OGP, DDGP and DM) $W_{A}$ of aqueous surfactant solutions to PE is equal to that determined for sucrose ester ones but only if it was determined from the $\gamma_{LV}\cos\theta$ vs. $\gamma_{LV}$ relationship in the range of surfactant concentration corresponding to its unsaturated monolayer at the water–air interface. In the case of glucose-based surfactants $W_{A}$ changes if the concentration of surfactant is close to that corresponding to its saturated monolayer at the water–air interface. Lee [50] suggested that the surface tension of liquid remains constant during the contact with the solid surface. However, as it was stated earlier, the orientation of OGP, DDGP and DM molecules toward the PE–water interface can change and then differs from that toward the water–air interface. If so, the surface tension of the surfactant solution drop settled on the PE surface at the parallel orientation of the glucose-based surfactant molecule can be different from the perpendicular one. In such a case Equation (19) should be written as follows [16]:

(22) $$W_{A}=\gamma_{LV}^{*}+\gamma_{SV}-\gamma_{SL}$$

where $\gamma_{LV}^{*}$ is the surfactant solution surface tension changed due to of the surfactant molecule orientation change.

Considering Equations (3), (20) and (22) for PE we can write:

(23) $$\gamma_{LV}(\cos\theta +\kappa )=2\sqrt{\gamma_{S}^{LW}\gamma_{L}^{LW}}$$

where $\gamma_{LV}^{*}=\kappa \gamma_{LV}$.

Next using the Lifshitz-van der Waals component value of PE and water surface tension (Table 2) as well as the surface tension [29,30] and contact angle of DDGP and DM aqueous solution (Figure 2 and Figure 3) the *κ* values were calculated from Equation (23) and are presented in Figure S27. Accordingly, from this figure the *κ* value changes with the surfactant concentration and is higher than unity. It proves that the solid surface tension influences on the solution surface tension as well as on the orientation of surfactant molecules at the PE–water interface during the PE wettability process causing the decrease of $W_{A}$ at the concentration of surfactant in solution corresponding to its saturated monolayer at the water–air interface.

In the case of PMMA, nylon 6 and quartz the $W_{A}$ values (Figures S24–S26) were calculated from Equations (20) and (5) or (7). It was found that when for $W_{A}$ calculation in Equation (5) the $\pi_{e}$ values were used, a good agreement between the $W_{A}$ values from Equations (5) and (20) was observed but only in the range of concentrations of surfactant corresponding to its unsaturated monolayer at the water–air interface. The best agreement is found (in the whole range of concentrations of surfactant in solution) if instead of the $\pi_{e}$ values in Equation (5) the $\pi_{e}$/2 ones were used. This means that it is possible to predict the $W_{A}$ to PMMA, nylon 6 and quartz surface using the Young-Dupré, Neuman at al. and van Oss et al. equations [1,32,33,34,35,40,41]. From Figures S24–S26 it can be seen that $W_{A}$ for mono- and bipolar solids changes with the surfactant concentration in the solution. This suggests that a part of $W_{A}$ relates to the surface coverage by surfactant molecules and adsorption layer formation at the solid–water interface.

For further considerations of adsorption of glucose and sucrose-based surfactants at the solid–water interface, the changes of the standard Gibbs free energy of adsorption of studied surfactants on the polymers and quartz surface should be determined.

### 2.7. Efficiency of Sucrose Acid Esters Adsorption at the Solid–Water Interface

The adsorption isotherms should be reflected by the standard Gibbs free energy of adsorption of studied surfactants on the polymers and quartz surface ($\Delta G_{ads}^{0}$). There are many approaches which can be used for determination of $\Delta G_{ads}^{0}$ [1,51,52,53,54,55,56]. Among others, the Langmuir equation modified by de Boer can be applied [1,53]:

(24) $$\frac{A_{SL}^{0}}{A_{SL}-A_{SL}^{0}}\exp\frac{A_{SL}^{0}}{A_{SL}-A_{SL}^{0}}=\frac{C_{S}}{\omega }\exp\left(\frac{\Delta G_{ads}^{0}}{RT}\right)$$

where $A_{SL}^{0}$ is the area occupied by the surfactant molecule at the solid–water interface, ω is the number of water moles in 1 dm^3^. To calculate $\Delta G_{ads}^{0}$ from Equation (24) the values of $A_{SL}^{0}$ for a given surfactant must be known. This value was determined earlier from Equation (17) (Table 3). The $\Delta G_{ads}^{0}$ values can be also determined from the linear form of the Langmuir equation [1]:

(25) $$\frac{C_{S}}{\Gamma_{SL}}=\frac{C_{S}}{\Gamma_{SL}^{\max}}+\frac{a_{S}}{\Gamma_{SL}^{\max}}$$

The obtained $\Delta G_{ads}^{0}$ values of all studied surfactants are presented in Table 5 as well as in Figures S28–S32. It follows from these figures that the $\Delta G_{ads}^{0}$ values calculated from Equation (24) are constant only in the range of concentration of surfactant in solution corresponding to its unsaturated monolayer at the water–air interface [29,30].

The $\Delta G_{ads}^{0}$ value changes with the concentration of surfactant in solution are different for different solids (Figures S28–S32) and result from the intermolecular interactions between the surfactant molecules in the solid–water adsorption layer.

In the case of adsorption of surfactants at the solid–water interface Gu and Zhu [54,55,56] suggested the following general adsorption isotherm equation which in the logarithmic form is as follows:

(26) $$\log\left(\frac{\Gamma_{SL}}{\Gamma_{SL}^{\infty }-\Gamma_{SL}}\right)=\log K+n\log C_{S}{}_{}$$

where *K* is the equilibrium constant of the surface aggregation process and *n* is the average aggregation number of the surface aggregates.

A plot of $\log\left(\frac{\Gamma_{SL}}{\Gamma_{SL}^{\infty }-\Gamma_{SL}}\right)$ versus $C_{S}$ permits evaluation of *K* and *n* when the data give a straight line. When *n* =1 then *K* = 1/*a* and Equation (25) becomes the Langmuir adsorption isotherm one. The *a* constant in the Langmuir equation at 293 K satisfies the relationship [1]:

(27) $$a=55.4\exp\frac{\Delta G_{ads}^{0}}{RT}$$

where $\Delta G_{ads}^{0}$ is the standard Gibbs free energy of adsorption.

It proves that there is the linear dependence between $\log\left(\frac{\Gamma_{SL}}{\Gamma_{SL}^{\infty }-\Gamma_{SL}}\right)$ and $C_{S}$ in the range of concentration of surfactant corresponding to its unsaturated monolayer at the water–air interface for which n is close to unity. Thus, it was possible to calculate $\Delta G_{ads}^{0}$ of the studied surfactants for PTFE, PE, PMMA, nylon 6 and quartz from Equation (27) (Table 5). It was found that the values of $\Delta G_{ads}^{0}$ for OGP, DDGP, DM, SMD and SML calculated from Equation (27) are somewhat lower that those obtained from Equation (24) (Table 5).

From our previous studies [19] it can also be seen that it is possible to determine the $\Delta G_{ads}^{0}$ for some apolar polymers from the following equation:

(28) $$\Delta G_{ads}^{0}=RT\left(\ln CMC-\ln\omega \right)-\frac{\gamma_{LV}\cos\theta_{S}-\gamma_{W}\cos\theta_{W}}{\Gamma_{SL}^{\max}}$$

where: $\theta_{S}$ and $\theta_{W}$ are the contact angles of solution at the CMC and water, respectively.

Thus, in the paper $\Delta G_{ads}^{0}$ values for the studied systems from $\theta_{S}$, $\theta_{W}$ and CMC of particular surfactants values (Table 1) were also determined from Equation (28) and are presented in Table 5.

From the comparison of the $\Delta G_{ads}^{0}$ values determined from Equations (24) and (27) to those determined from Equation (28), it can be stated that only in the case of hydrophobic low-energetic polymers it is possible to determine the $\Delta G_{ads}^{0}$ at the solid–water interface based on the CMC value as well as the contact angle and surface tension values at this concentration. $\Delta G_{ads}^{0}$ calculated from Equation (28) for polar polymers and quartz give much higher values than those calculated from other equations, thus they cannot be treated as real ones. This results from the fact that in the case of polar polymers and quartz the solid surface tension is changed during the wettability process because of the surfactant film formation and in such a case Equation (28) cannot be applied.

## 3. Conclusions

From the studied polymers and quartz wettability considerations it can be seen that:

It is possible to predict the contact angle of the aqueous solutions of sucrose fatty acid ester and some other sugar-based surfactants on the studied polymers and quartz based on the new Lifshitz-van der Waals component of the water surface tension as well as the components and parameters of the surfactant and solid surface tension.

The orientation, packing and properties of adsorption layer of all studied sugar surfactants at the PTFE–water interface is similar to that at the water–air one.

The orientation of sugar surfactant molecules at the polar polymer or quartz–water interface is rather parallel which means that the surfactant concentration at these interfaces as well as the properties of the surfactant adsorption layer are different from those at the water–air and PTFE–water ones.

The critical surface tension of solid wetting is equal to that of solid surface tension if its value is strictly equal to the new Lifshitz-van der Waals component value of the water surface tension.

It is possible to predict the work of adhesion of studied surfactants to the solid surface using the Young-Dupré, Neuman at al. and van Oss et al. equations.

A part of this work relates to the surface coverage by surfactant molecules and adsorption layer formation at the solid–water interface.

In the case of hydrophobic low-energetic polymers, it was possible to determine the Gibbs free energy of adsorption at the solid–water interface based on the CMC value and the contact angle and surfactant solution surface tension values at the CMC.

Considering the antimicrobial properties of sucrose fatty acid esters and the fact that their adsorption layer at the polymer–water interface is closely packed, they can be potentially applied as polymer surface wettability modifiers for their biomedical applications.

## 4. Experimental

### 4.1. Materials

For the contact angle measurements the aqueous solutions of sucrose capric acid ester (SMD) (purity > 97%), sucrose lauric acid ester (SML) (purity > 97), *n*-dodecyl-*β*-d-maltoside (DM) (purity > 98%), *n*-octyl-*β*-d-glucopyranoside (OGP) (purity > 98%) and *n*-dodecyl-*β*-d-glucopyranoside (DDGP) (purity > 98%) (purchased from the Sigma-Aldrich, Poznań, Poland) as well as polytetrafluoroethylene (PTFE), polyethylene (PE), polymethyl methacrylate (PMMA), polyamide (nylon 6) (purchased from the Mega-Tech, Grodzisk Mazowiecki, Poland) and quartz (purchased from Conductance, Ostrów Wielkopolski, Poland) were used. The solids surface was prepared in an appropriate way before the contact angle measurements. The aqueous solutions of studied surfactants were prepared using doubly distilled and deionized water (Destamat Bi18E). The purity of water was additionally controlled by the surface tension and contact measurements before preparing the solutions.

### 4.2. Contact Angle Measurements

The measurements of the advancing contact angles of aqueous solutions of SMD, SML, OGP, DDGP and DM on the PTFE, PE, PMMA, nylon 6 and quartz surface were made using the sessile drop method and the DSA30 measuring system (Krüss), in a thermostated chamber at 293 ± 0.1 K. The chamber of apparatus was saturated by the vapor of a given liquid for which the contact angle was measured by introducing a cell filled with a given liquid three hours before measurements. The contact angle for a given solution was measured for at least 30 drops. For all the contact angle measurements the drops of 7 µL volume were used. Good reproducibility was found for the contact angle measurements. The standard deviation for each set of values was less than 1.2°.

Before the contact angle measurements, the polymer plates (PTFE, PE, PMMA, nylon 6) were polished with a light pressure on a Buchler polishing wheel using a clean, dry, silk polishing cloth. These plates were washed sequentially with a detergent and next with methanol, placed twice in an ultrasonic bath in the Milli-Q water for 15 min. and dried in the desiccator with a molecular sieve at room temperature. The quartz plates were cleaned with soapy water, washed many times in distilled water and placed in the ultrasonic bath for 15 min. This procedure was repeated twice for each plate. Then the plates were dried and placed in the desiccator with molecular sieve.

The surface topography of polymers and quartz plates was examined using an optical profilometer (Contour GT, Veeco) (Scheme S1) and atomic force microscopy (AFM) (Nanoscope 3, VEECO). The plate with the smallest roughness was used for the contact angle measurements. Additionally, the surface chemistry of solid surfaces was checked using the Fourier transform infrared (FT-IR) spectroscopy. The FT-IR spectrum was recorded on a 1725X Perkin-Elmer spectrophotometer at room temperature with a resolution of 4 cm^−1^.

## Figures and Tables

**Table 1:** The values of critical micelle concentration (CMC) and contact angle at CMC ($\theta_{CMC}$) of studied surfactants determined from the contact angle isotherms.

Surfactant	PTFE		PE		PMMA		Nylon 6		Quartz	
	CMC [mM]	$\theta_{CMC}$	CMC [mM]	$\theta_{CMC}$	CMC [mM]	$\theta_{CMC}$	CMC [mM]	$\theta_{CMC}$	CMC [mM]	$\theta_{CMC}$
OGP	25.70	60.00	19.95	67.90	28.80	39.00	25.37	34.60	25.37	22.00
DDGP	0.12	64.48	0.14	56.00	0.09	42.48	0.09	45.00	0.09	28.20
DM	0.18	73.42	0.19	48.98	0.19	47.91	0.19	47.91	0.18	28.80
SMD	2.03	72.00	2.03	46.00	2.00	48.20	2.00	48.20	2.00	28.00
SML	0.33	76.40	0.34	52.00	0.32	50.22	0.32	50.22	0.32	30.00

**Table 2:** The values of water, solid and surfactant surface tension ($\gamma_{}$), the Lifshitz-van der Waals ($\gamma_{}^{LW}$) and acid-base component, as well as the electron-donor ($\gamma_{}^{-}$) and electron-acceptor ($\gamma_{}^{+}$) parameters of this tension [36,37,38].

	$\gamma^{LW}$ [mN/m]	$\gamma^{AB}$ [mN/m]	$\gamma^{+}$ [mN/m]	$\gamma^{-}$ [mN/m]	$\gamma_{}$ [mN/m]
Water	26.85	45.95	22.975	22.975	72.80
PTFE	20.24	0.00	0.00	0.00	20.24
PE	33.71	0.00	0.00	0.00	33.71
PMMA	41.28	0.00	0.00	7.28	41.28
Nylon 6	36.44	2.57	0.15	11.10	39.01
Quartz	38.07	9.63	1.61	14.36	47.70
OGP, head OGP, tail	33.71 21.80	5.66 -	0.14 -	57.55 -	39.37 21.80
DDGP, head DDGP, tail	33.71 25.08	5.66 -	0.14 -	57.55 -	39.37 25.08
DM, head DM, tail	32.02 25.08	8.38 -	0.30 -	58.27 -	40.40 25.08
SMD, head SMD, tail	33.15 22.91	6.75 -	0.20 -	57.23 -	39.90 22.91
SML, head SML, tail	30.87 25.05	9.80 -	0.42 -	57.50 -	40.67 25.05

**Table 3:** The values of maximal Gibbs surface excess concentration ($\Gamma_{SL}^{\max}$), minimal area ($A_{SL}^{\min}$), maximal possible Gibbs surface excess concentration ($\Gamma_{SL}^{\infty }$), excluded area ($A_{SL}^{0}$), theoretical area ($A_{SL(theoretical)}^{0}$) and theoretical Gibbs surface excess concentration ($\Gamma_{SL(theoretical)}^{\infty }$) of OGP, DDGP, DM, SMD and SML at their perpendicular and parallel (with one or two sugar units of surfactant disaccharide polar part) orientation toward the solid–water interface.

Surfactant	Solid	$\Gamma_{SL}^{\max}$ ×10^−6^ [mol/m^2^]	$A_{SL}^{\min}$ [Å^2^]	$\Gamma_{SL}^{\infty }$ ×10^−6^ [mol/m^2^]	$A_{SL}^{0}$ [Å^2^]	$A_{SL(theoretical)}^{0}$ [Å^2^]			$\Gamma_{SL(theoretical)}^{\infty }$ ×10^−6^ [mol/m^2^]		
						a	b		c	d	
							1	2		1	2
OGP	PMMA	1.80	92.24	2.18	76.12	35.05	84.78	_	4.74	1.96	_
	nylon 6	1.50	110.69	1.96	84.70						
	Quartz	1.04	159.65	1.99	83.50						
DDGP	PTFE	4.27	38.88	4.52	36.74	35.05	107.87	_	4.74	1.54	_
	PE	3.41	48.69	4.59	36.19						
	PMMA	1.53	108.52	1.98	83.85						
	nylon 6	1.40	118.65	1.52	109.00						
	Quartz	0.90	184.48	1.46	113.98						
DM	PTFE	3.30	50.31	4.56	36.42	70.10	107.87	142.92	2.37	1.54	1.16
	PE	3.21	51.72	4.61	36.00						
	PMMA	1.51	109.95	2.71	61.30						
	nylon 6	1.16	143.13	1.52	109.00						
	Quartz	0.91	182.45	1.60	103.89						
SMD	PTFE	3.12	53.21	4.65	35.67	60.93	106.99	132.87	2.72	1.55	1.25
	PE	3.08	53.91	4.49	37.00						
	PMMA	1.53	108.52	2.26	73.50						
	nylon 6	1.25	132.82	1.58	105.33						
	Quartz	0.85	195.33	1.55	107.00						
SML	PTFE	3.11	53.39	4.50	36.86	60.93	108.14	134.02	2.72	1.54	1.24
	PE	3.03	54.80	4.50	36.86						
	PMMA	1.47	112.95	2.75	60.31						
	nylon 6	1.21	137.22	1.52	109.51						
	Quartz	0.81	204.98	1.55	107.28						

A—$A_{SL(theoretical)}^{0}$, perpendicular orientation; b—$A_{SL(theoretical)}^{0}$, parallel orientation: 1—with one sugar unit of surfactant polar part, 2—with two sugar units of surfactant polar part; c—$\Gamma_{SL(theoretical)}^{\infty }$, perpendicular orientation; d—$\Gamma_{SL(theoretical)}^{\infty }$, parallel orientation: 1—with one sugar unit of surfactant polar part, 2—with two sugar units of surfactant polar part.

**Table 4:** The values of the solid critical surface tension of wetting ($\gamma_{C}$).

Surfactant	Solid	$\gamma_{C}$ [mN/m]	
		$\gamma_{LV}\cos\theta$ vs. $\gamma_{LV}$	cosθ vs. $\gamma_{LV}$
OGP	PMMA	28.02	-
	nylon 6	29.19	-
	Quartz	-	24.52
DDGP	PTFE	23.27	18.29
	PE	30.36	18.29
	PMMA	27.96	-
	nylon 6	29.74	-
	Quartz	-	23.55
DM	PTFE	23.03	17.46
	PE	30.15	27.07
	PMMA	28.40	-
	nylon 6	29.44	-
	Quartz	-	23.85
SMD	PTFE	23.20	16.46
	PE	30.07	27.89
	PMMA	28.58	-
	nylon 6	29.54	-
	Quartz	-	25.09
SML	PTFE	22.96	15.03
	PE	30.08	27.61
	PMMA	28.52	-
	nylon 6	29.27	-
	Quartz	-	25.08

**Table 5:** The values of standard Gibbs free energy ($\Delta G_{ads}^{0}$) of OGP, DDGP, DM, SMD and SML adsorption at the solid–water interface.

Surfactant	$\Delta G_{ads}^{0}$ [kJ/mol]				
OGP		Equation (24)	Equation (25)	Equation (27)	Equation (28)
	PMMA	−29.99	−32.21	−31.11	−20.30
	nylon 6	−30.20	−32.40	−31.01	−18.52
	Quartz	−29.58	−33.27	−31.89	−6.75
DDGP	PTFE	−42.61	−43.37	−42.46	−41.65
	PE	−42.60	−44.30	−43.62	−40.86
	PMMA	−44.03	−46.15	−45.40	−35.08
	nylon 6	−45.25	−46.64	−45.96	−30.51
	Quartz	−44.81	−47.36	−46.69	−18.72
DM	PTFE	−39.17	−42.33	−41.72	−41.77
	PE	−39.48	−42.73	−42.34	−41.80
	PMMA	−39.94	−44.76	−44.63	−33.45
	nylon 6	−41.52	−45.60	−45.92	−29.86
	Quartz	−40.63	−45.81	−46.23	−21.48
SMD	PTFE	−34.54	−37.39	−36.92	−36.80
	PE	−34.87	−37.80	−36.68	−37.00
	PMMA	−35.47	−38.69	−37.49	−27.47
	nylon 6	−36.08	−38.95	−37.90	−23.94
	Quartz	−31.59	−33.99	−33.80	−15.15
SML	PTFE	−38.91	−41.20	−40.93	−40.59
	PE	−38.97	−42.19	−41.71	−40.98
	PMMA	−39.39	−43.50	−43.70	−32.18
	nylon 6	−40.76	−43.92	−41.55	−28.53
	Quartz	−39.86	−44.41	−45.21	−20.26

## Supplementary Materials

The supplementary materials are available online.
