# Supplementary material for: Solid Wettability Modification via Adsorption of Antimicrobial Sucrose Fatty Acid Esters and Some Other Sugar-Based Surfactants
Source: Molecules. 2018 Jul 1;23(7):1597. doi: 10.3390/molecules23071597 (PMC6100448; doi:10.3390/molecules23071597)
Supplement: Supplementary file 1 [file molecules-23-01597-s001.pdf]

SUPPLEMENTARY MATERIAL

SOLID WETTABILITY MODIFICATION VIA ADSORPTION OF ANTIMICROBIAL SUCROSE FATTY ACID ESTERS AND SOME OTHER SUGAR-BASED SURFACTANTS

JOANNA KRAWCZYK *

Department of Interfacial Phenomena, Faculty of Chemistry, Maria Curie-Skłodowska University, Maria Curie-Skłodowska Sq. 3, 20-031 Lublin, Poland

*Correspondence: *j.krawczyk@poczta.umcs.lublin.pl*; Tel.: +48 (81) 537-56-03

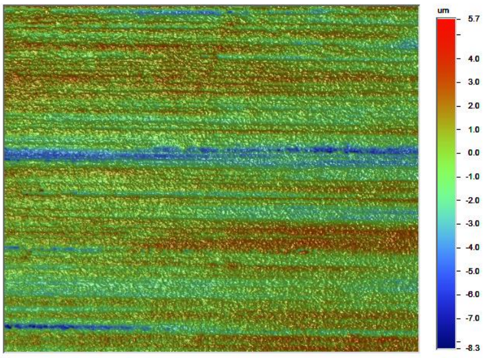

PTFE (Ra=1.03 µm, Rq=1.32 µm, sampling 1.97 µm)

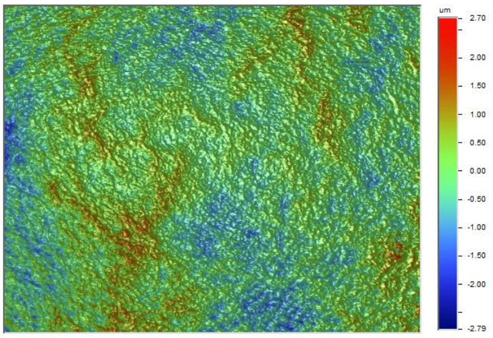

PE (Ra=384.14 nm, Rq=482.08 nm, sampling 1.97 nm)

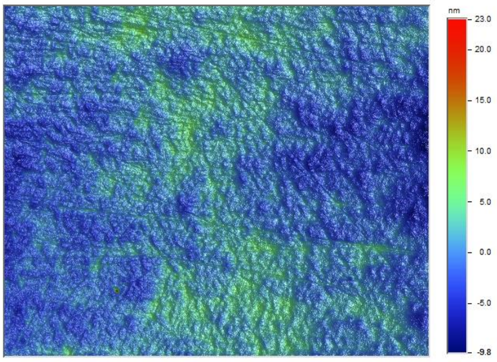

PMMA (Ra=1.94 nm, Rq=2.40 nm, sampling 1.97 µm)

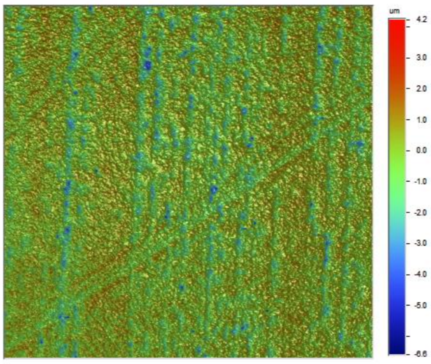

PMMA (Ra=544.00 nm, Rq=474.37 nm, sampling 1.97 µm)

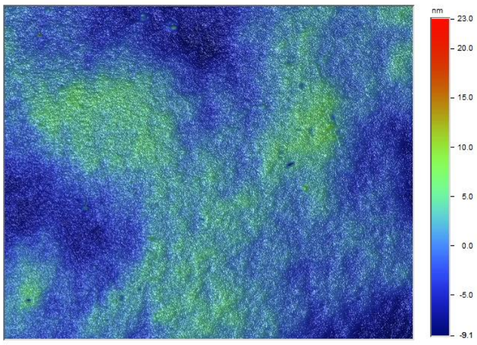

Quartz (Ra=2.40 nm, Rq=2.98 nm), sampling 1.97 µm

Scheme S1. Profilometer analysis of the studied solids surface.

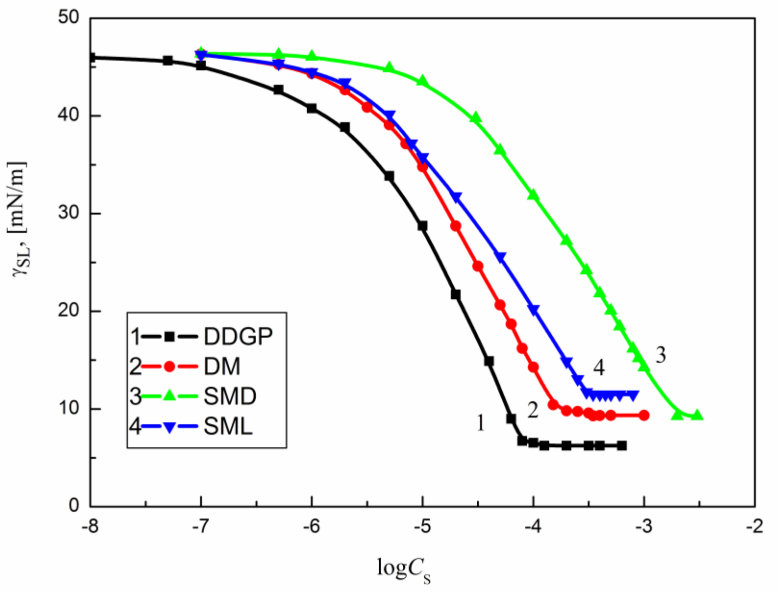

Fig. S1. A plot of the PTFE-water interface tension (γ_{SL}) of the aqueous solutions of DDDGP (curve 1), DM (curve 2), SMD (curve 3) and SML (curve 4) vs. the logarithm of surfactant concentration (log C_S).

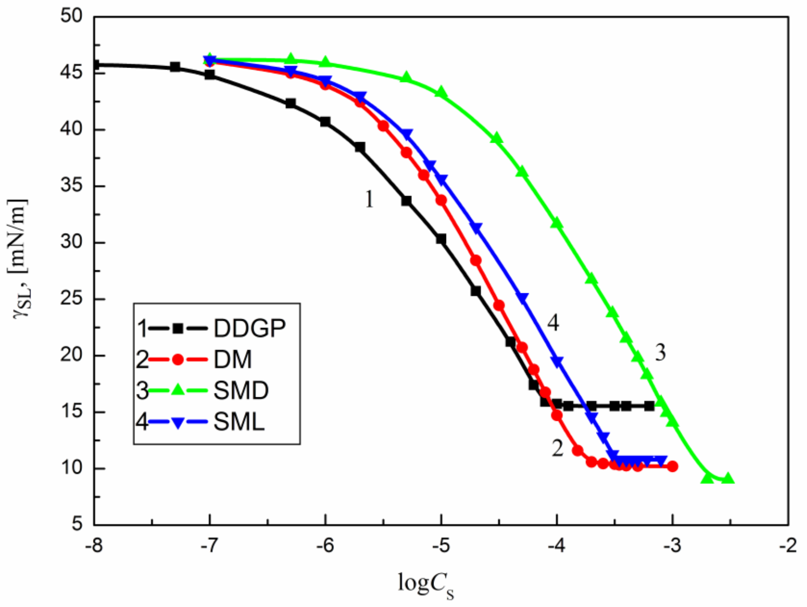

Fig. S2. A plot of the PE-water interface tension (γ_{SL}) of the aqueous solutions of DDDGP (curve 1), DM (curve 2), SMD (curve 3) and SML (curve 4) vs. the logarithm of surfactant concentration (log C_S).

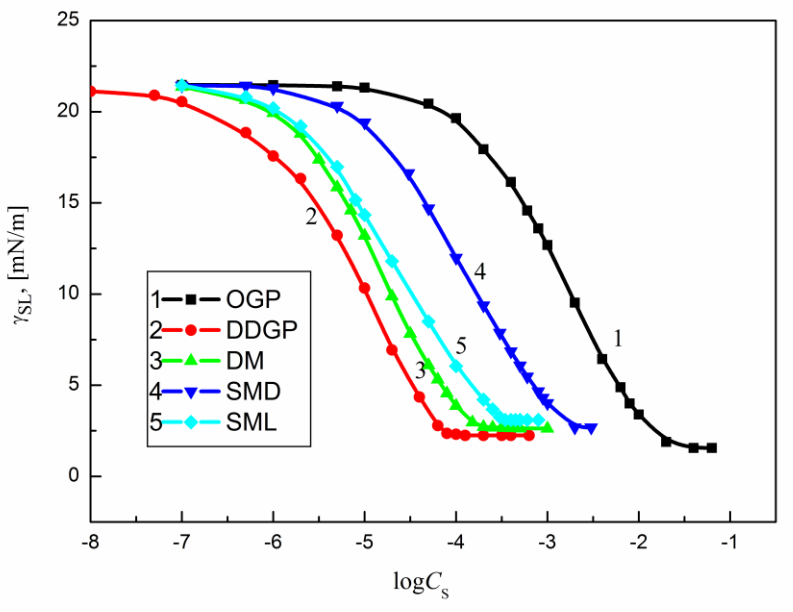

Fig. S3. A plot of the PMMA-water interface tension (γ_{SL}) of the aqueous solutions of OGP (curve 1), DDGP (curve 2), DM (curve 3), SMD (curve 4) and SML (curve 5) vs. the logarithm of surfactant concentration (log C_S).

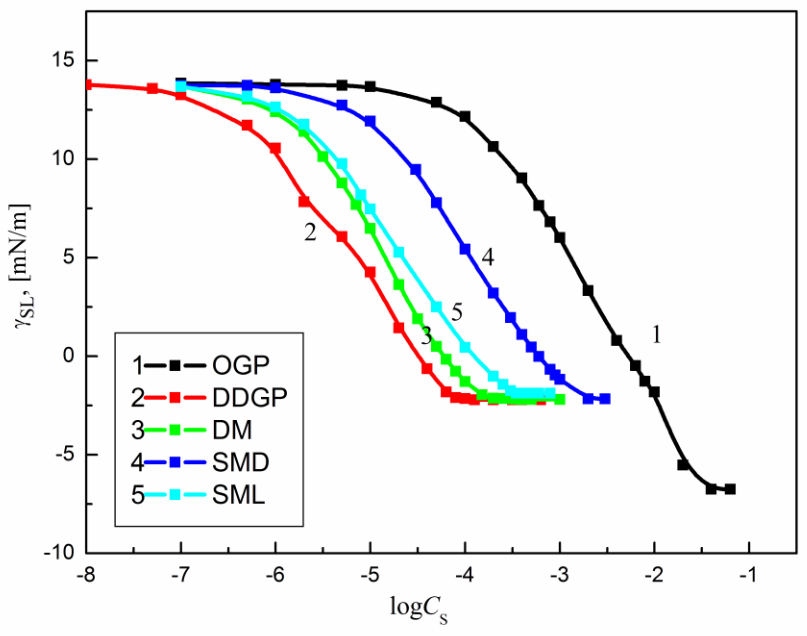

Fig. S4. A plot of the nylon 6-water interface tension (γ_{SL}) of the aqueous solutions of OGP (curve 1), DDGP (curve 2), DM (curve 3), SMD (curve 4) and SML (curve 5) vs. the logarithm of surfactant concentration (log C_S).

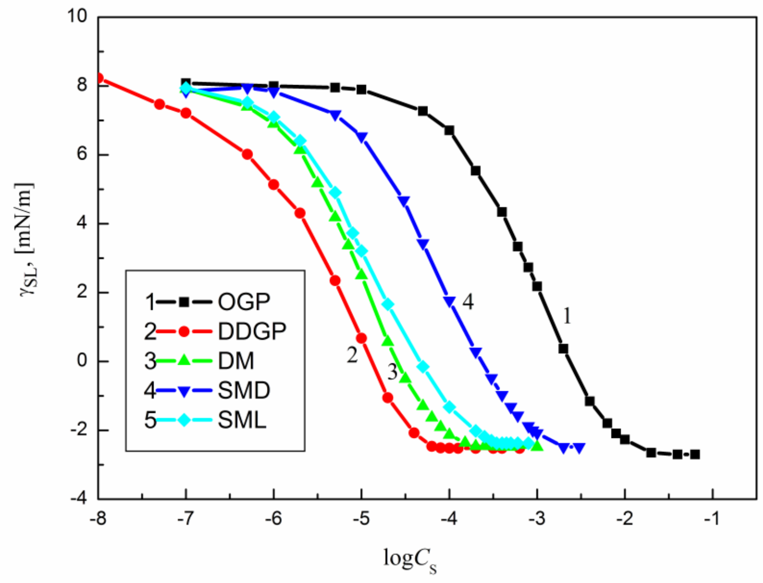

Fig. S5. A plot of the quartz-water interface tension (γ_{SL}) of the aqueous solutions of OGP (curve 1), DDGP (curve 2), DM (curve 3), SMD (curve 4) and SML (curve 5) vs. the logarithm of surfactant concentration (log C_S).

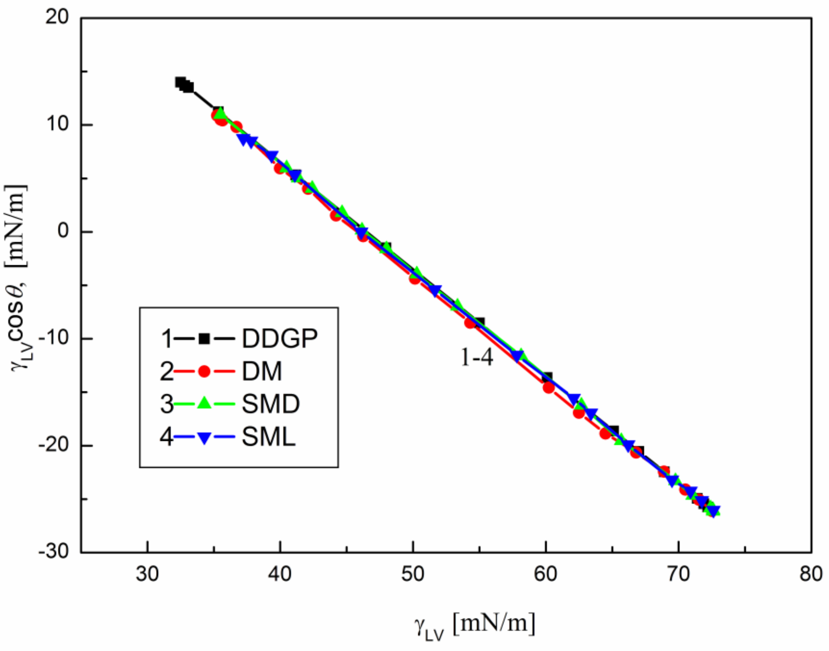

Fig. S6. A plot of the adhesion tension ($\gamma_{LV}\cos\theta$) of the aqueous solutions of DDGP (curve 1), DM (curve 2), SMD (curve 3) and SML (curve 4) for the PTFE surface vs. the solution surface tension (γ_{LV}).

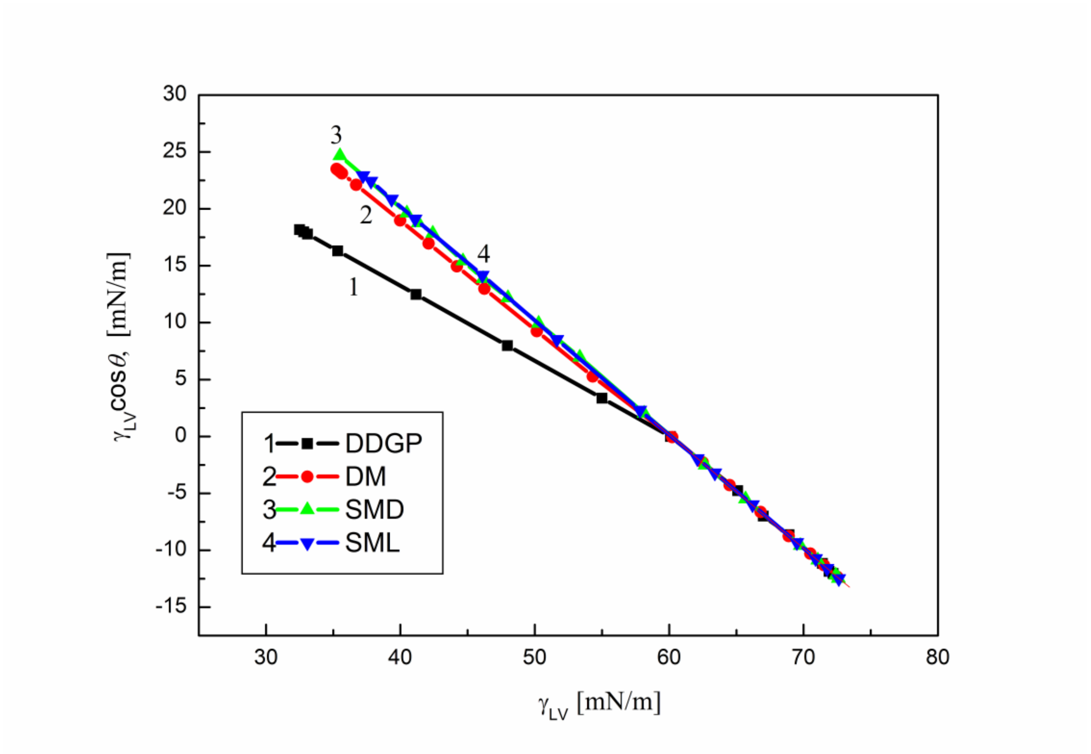

Fig. S7. A plot of the adhesion tension ($\gamma_{LV}\cos\theta$) of the aqueous solutions of DDGP (curve 1), DM (curve 2), SMD (curve 3) and SML (curve 4) for the PE surface vs. the solution surface tension (γ_{LV}).

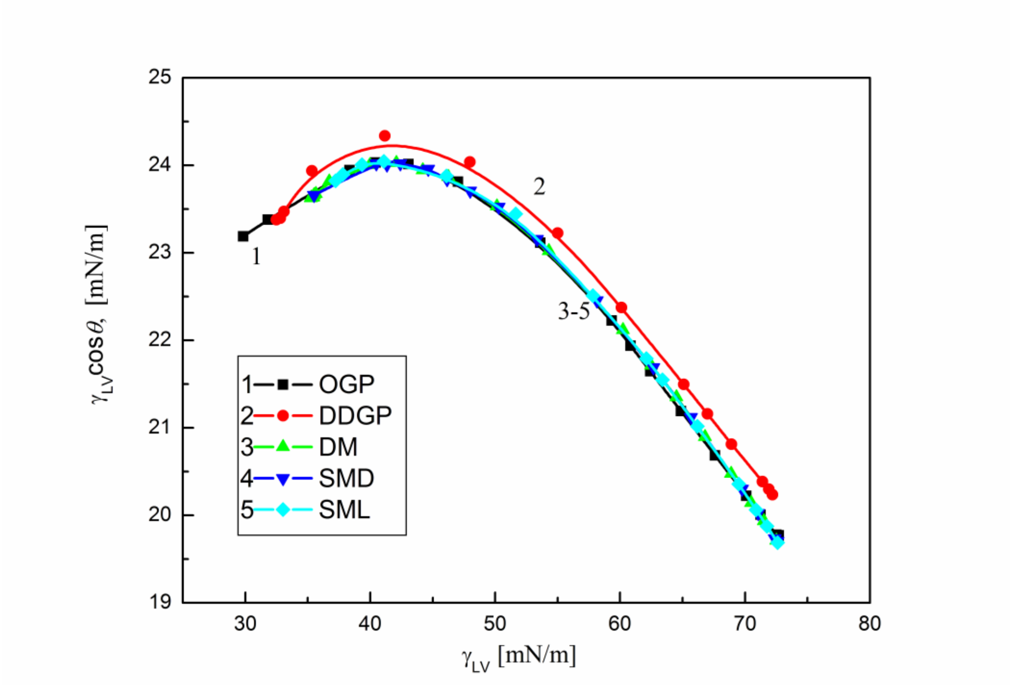

Fig. S8. A plot of the adhesion tension ($\gamma_{LV}\cos\theta$) of aqueous solutions of OGP (curve 1), DDGP (curve 2), DM (curve 3), SMD (curve 4) and SML (curve 5) for the PMMA surface vs. the solution surface tension (γ_{LV}).

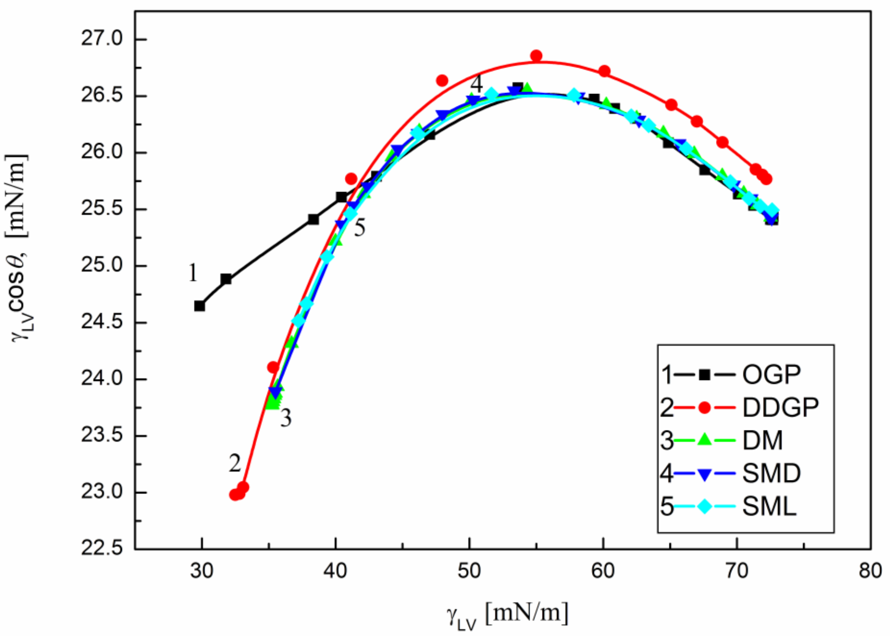

Fig. S9. A plot of the adhesion tension ($\gamma_{LV}\cos\theta$) of aqueous solutions of OGP (curve 1), DDGP (curve 2), DM (curve 3), SMD (curve 4) and SML (curve 5) for the nylon 6 surface vs. the solution surface tension (γ_{LV}).

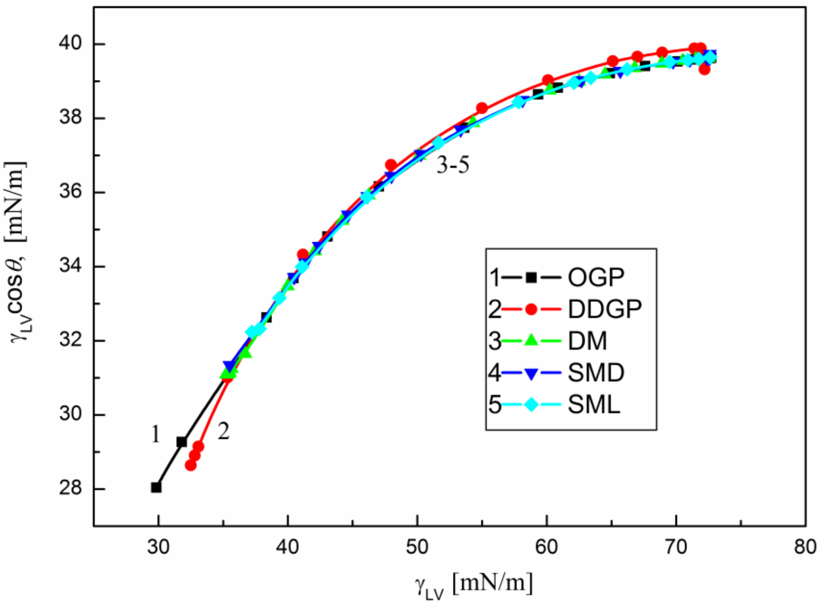

Fig. S10. A plot of the adhesion tension ($\gamma_{LV}\cos\theta$) of aqueous solutions of OGP (curve 1), DDGP (curve 2), DM (curve 3), SMD (curve 4) and SML (curve 5) for the quartz surface vs. the solution surface tension (γ_{LV}).

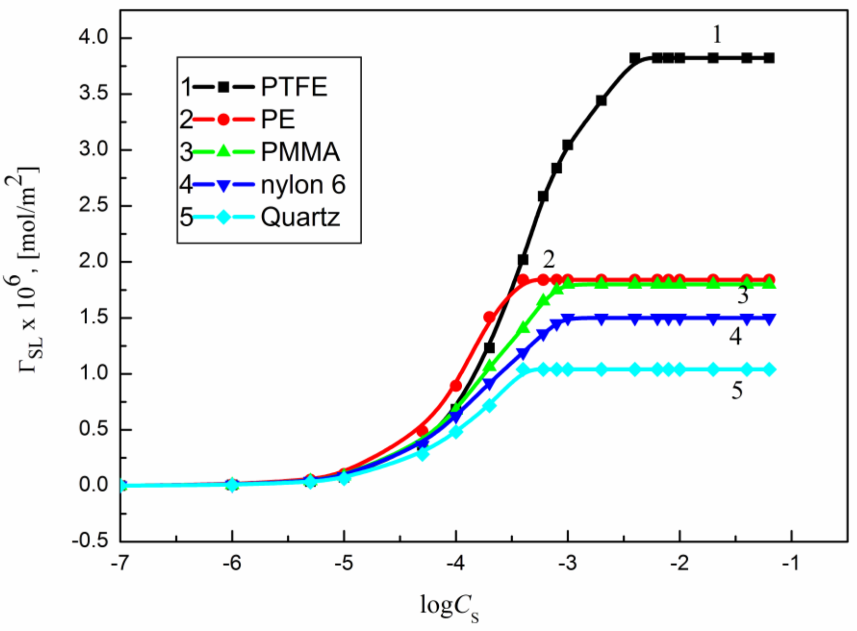

Fig. S11. A plot of the OGP Gibbs surface excess concentration (Γ_{SL}) (curves 1 – 5) at the PTFE, PE, PMMA, nylon 6 or quartz-water interface, respectively, vs. the logarithm of OGP concentration ($\log C_S$).

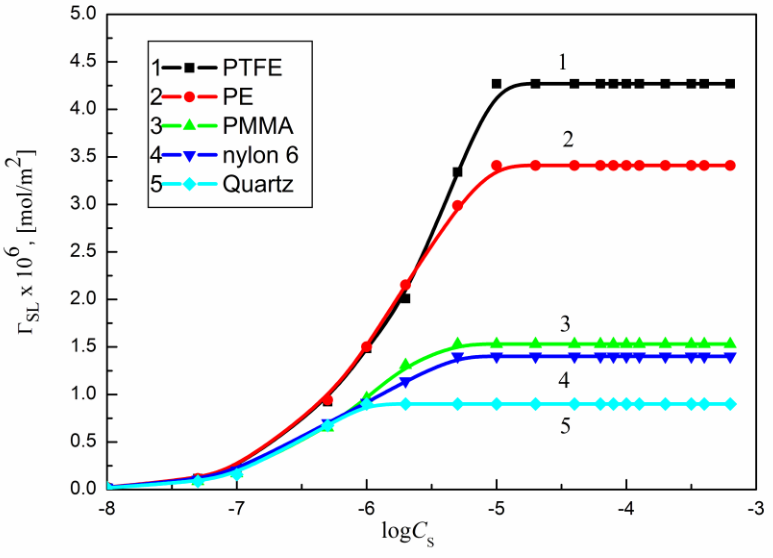

Fig. S12. A plot of the DDGP Gibbs surface excess concentration (Γ_{SL}) (curves 1 – 5) at the PTFE, PE, PMMA, nylon 6 or quartz-water interface, respectively, vs. the logarithm of DDGP concentration ($\log C_S$).

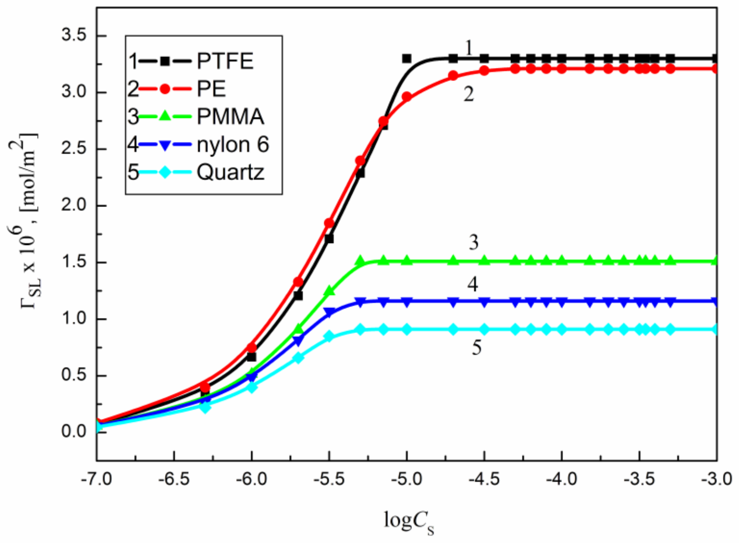

Fig. S13. A plot of the DM Gibbs surface excess concentration (Γ_{SL}) (curves 1 – 5) at the PTFE, PE, PMMA, nylon 6 or quartz-water interface, respectively, vs. the logarithm of DM concentration ($\log C_S$).

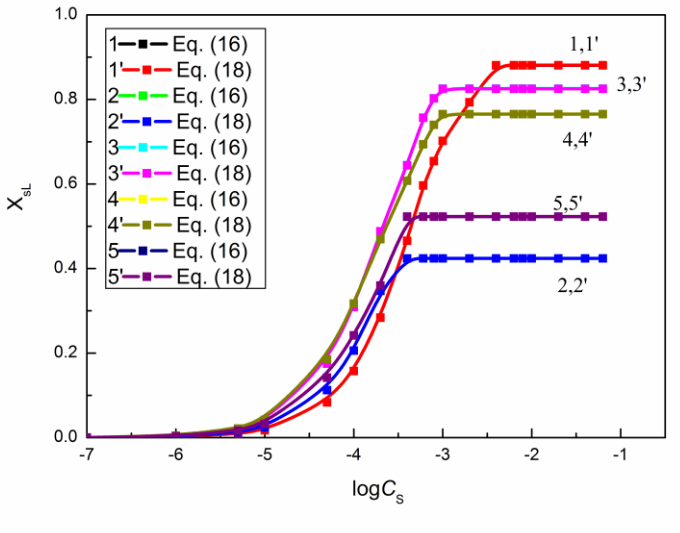

Fig. S14. A plot of the mole fraction of the area (X_{SL}) occupied by OGP at the PTFE-water (curve 1 and 1’), PE-water (curve 2 and 2’), PMMA-water (curve 3 and 3’), nylon 6 (curve 4 and 4’) and quartz-water (curve 5 and 5’) interface vs. the logarithm of surfactant concentration ($\log C_S$). Curves 1 – 5 and curves 1’ – 5’ correspond to the values calculated from Eq. (16) and Eq. (18), respectively.

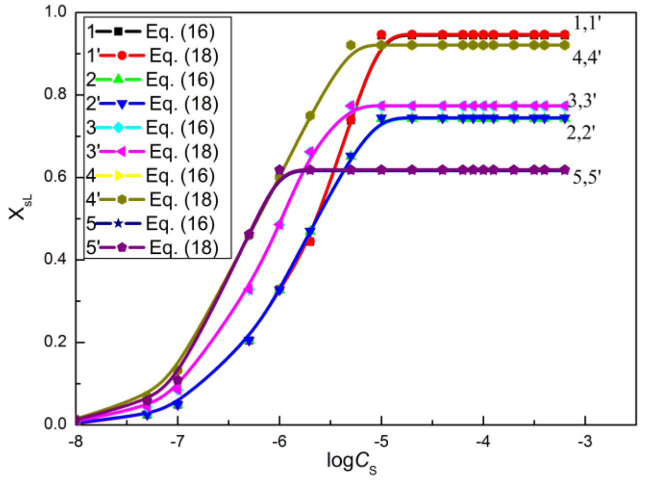

Fig. S15. A plot of the mole fraction of the area (X_{SL}) occupied by DDGP at the PTFE-water (curve 1 and 1'), PE-water (curve 2 and 2'), PMMA-water (curve 3 and 3'), nylon 6 (curve 4 and 4') and quartz-water (curve 5 and 5') interface vs. the logarithm of surfactant concentration ($\log C_S$). Curves 1 – 5 and curves 1' – 5' correspond to the values calculated from Eq. (16) and Eq. (18), respectively.

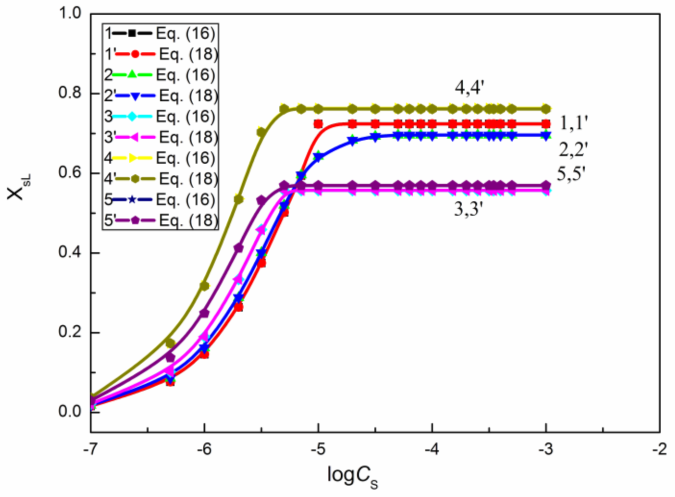

Fig. S16. A plot of the mole fraction of the area (X_{SL}) occupied by DM at the PTFE-water (curve 1 and 1'), PE-water (curve 2 and 2'), PMMA-water (curve 3 and 3'), nylon 6 (curve 4 and 4') and quartz-water (curve 5 and 5') interface vs. the logarithm of surfactant concentration ($\log C_S$). Curves 1 – 5 and curves 1' – 5' correspond to the values calculated from Eq. (16) and Eq. (18), respectively.

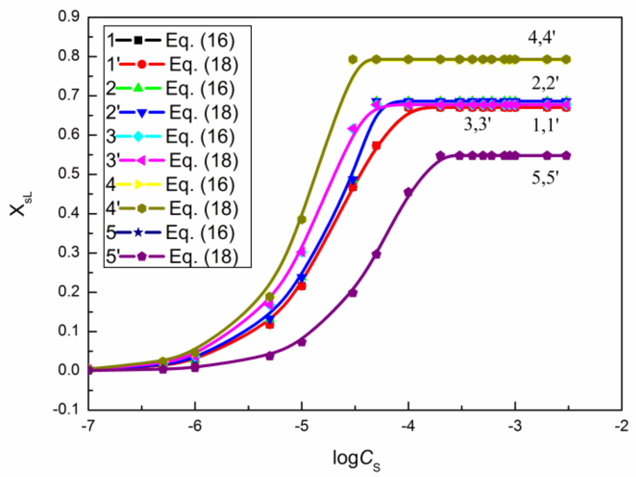

Fig. S17. A plot of the mole fraction of the area (X_{SL}) occupied by SMD at the PTFE-water (curve 1 and 1'), PE-water (curve 2 and 2'), PMMA-water (curve 3 and 3'), nylon 6 (curve 4 and 4') and quartz-water (curve 5 and 5') interface vs. the logarithm of surfactant concentration ($\log C_S$). Curves 1 – 5 and curves 1' – 5' correspond to the values calculated from Eq. (16) and Eq. (18), respectively.

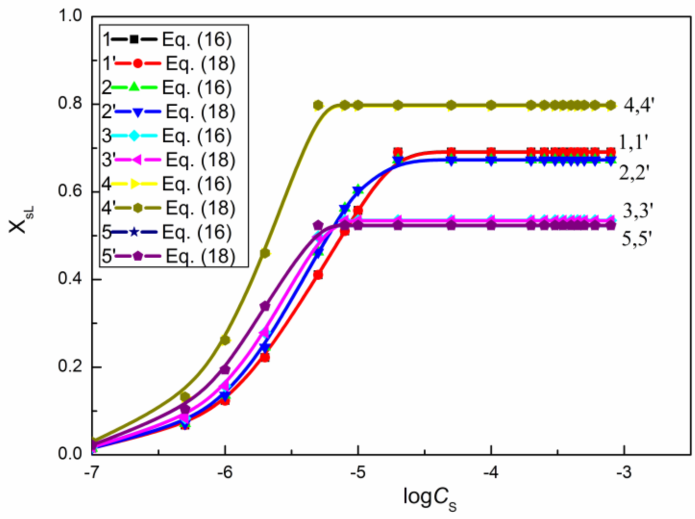

Fig. S18. A plot of the mole fraction of the area (X_{SL}) occupied by SML at the PTFE-water (curve 1 and 1'), PE-water (curve 2 and 2'), PMMA-water (curve 3 and 3'), nylon 6 (curve 4 and 4') and quartz-water (curve 5 and 5') interface vs. the logarithm of surfactant concentration ($\log C_S$). Curves 1 – 5 and curves 1' – 5' correspond to the values calculated from Eq. (16) and Eq. (18), respectively.

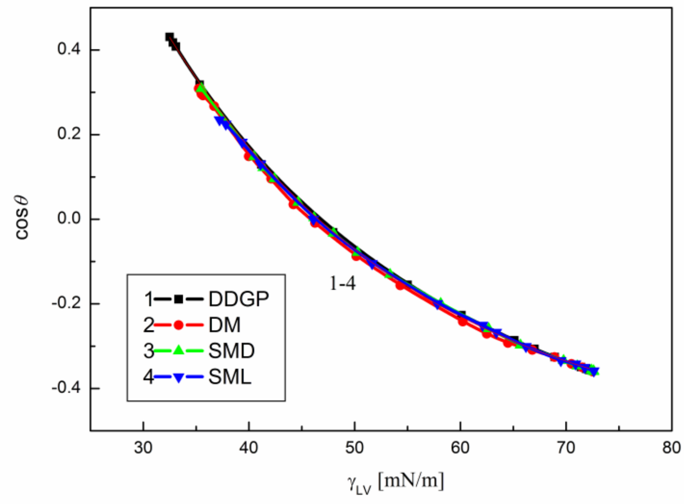

Fig. S19. A plot of the cosine of the contact angle ($\cos\theta$) of the aqueous solutions of DDGP (curve 1), DM (curve 2), SMD (curve 3) and SML (curve 4) for the PTFE surface vs. the surface tension (γ_{LV}).

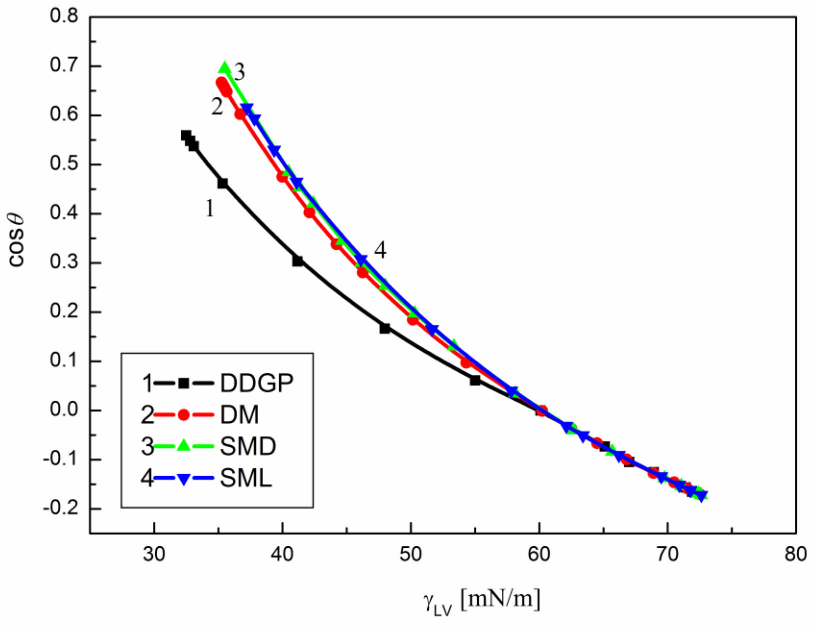

Fig. S20. A plot of the cosine of the contact angle ($\cos\theta$) of the aqueous solutions of DDGP (curve 1), DM (curve 2), SMD (curve 3) and SML (curve 4) for the PE surface vs. the surface tension (γ_{LV}).

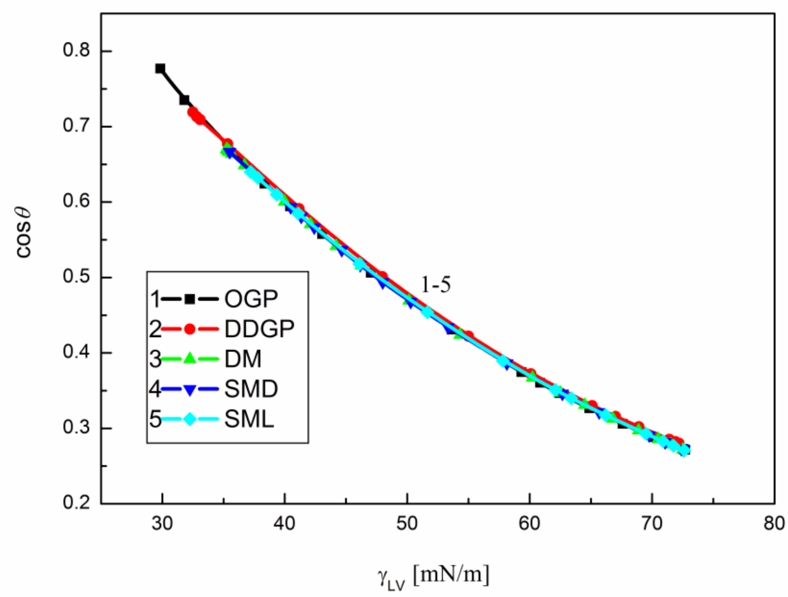

Fig. S21. A plot of the cosine of the contact angle ($\cos\theta$) of the aqueous solutions of OGP (curve 1), DDGP (curve2), DM (curve 3), SMD (curve 4) and SML (curve 5) for the PMMA surface vs. the surface tension (γ_{LV}).

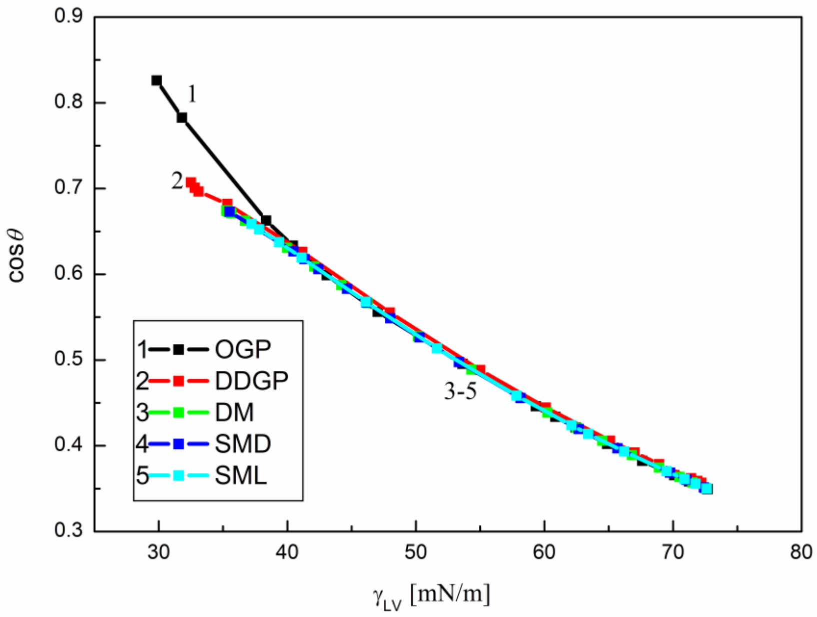

Fig. S22. A plot of the cosine of the contact angle ($\cos\theta$) of the aqueous solutions of OGP (curve 1), DDGP (curve2), DM (curve 3), SMD (curve 4) and SML (curve 5) for the nylon 6 surface vs. the surface tension (γ_{LV}).

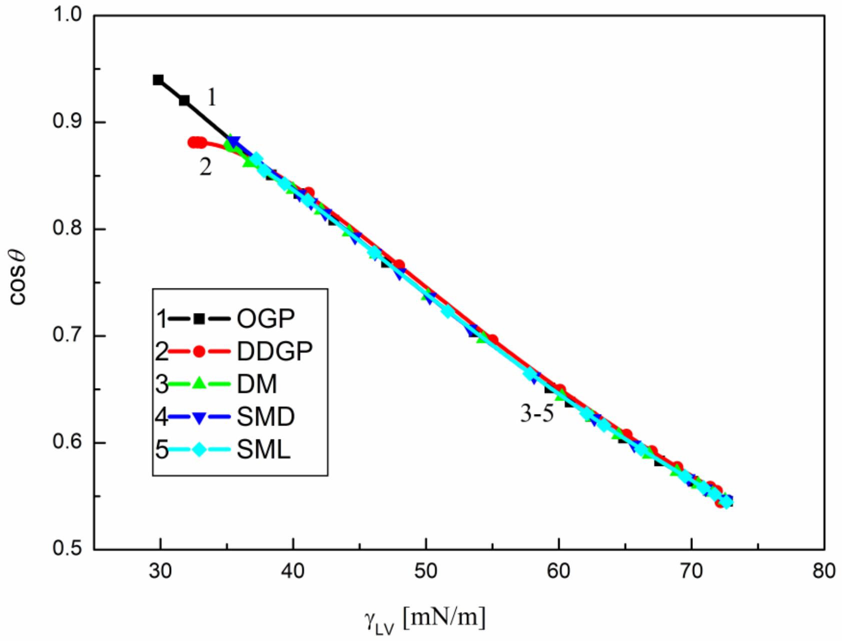

Fig. S23. A plot of the cosine of the contact angle ($\cos\theta$) of the aqueous solutions of OGP (curve 1), DDGP (curve2), DM (curve 3), SMD (curve 4) and SML (curve 5) for the quartz surface vs. the surface tension (γ_{LV}).

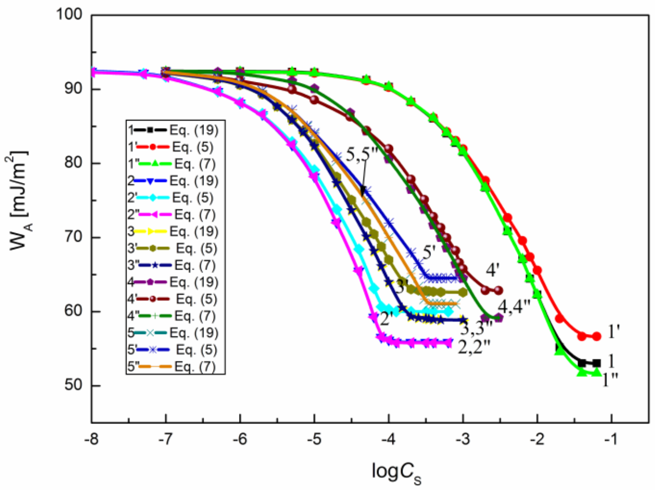

Fig. S24. A plot of the work of adhesion (W_A) of aqueous solution of OGP (curve 1, 1' and 1"), DDGP (curve 2, 2' and 2"), DM (curve 3, 3' and 3"), SMD (curve 4, 4' and 4") and SML (curve 4, 5' and 5") to the PMMA surface vs. the logarithm of surfactant concentration ($\log C_S$). Curves 1 – 5, curves 1' – 5' and curves 1"-5" correspond to the W_A values calculated from Eq. (19), Eq. (5) and Eq. (7), respectively.

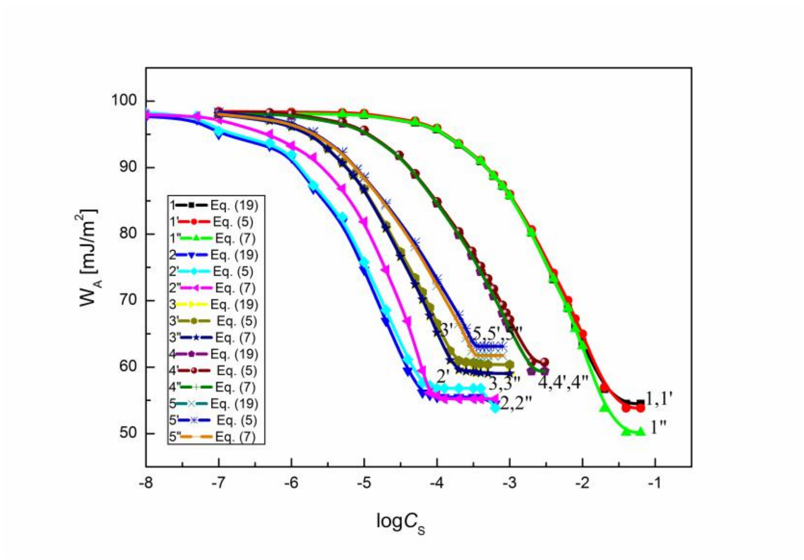

Fig. S25. A plot of the work of adhesion (W_A) of aqueous solution of OGP (curve 1, 1' and 1"), DDGP (curve 2, 2' and 2"), DM (curve 3, 3' and 3"), SMD (curve 4, 4' and 4") and SML (curve 4, 5' and 5") to the nylon 6 surface vs. the logarithm of surfactant concentration ($\log C_S$). Curves 1 – 5, curves 1' – 5' and curves 1"-5" correspond to the W_A values calculated from Eq. (19), Eq. (5) and Eq. (7), respectively.

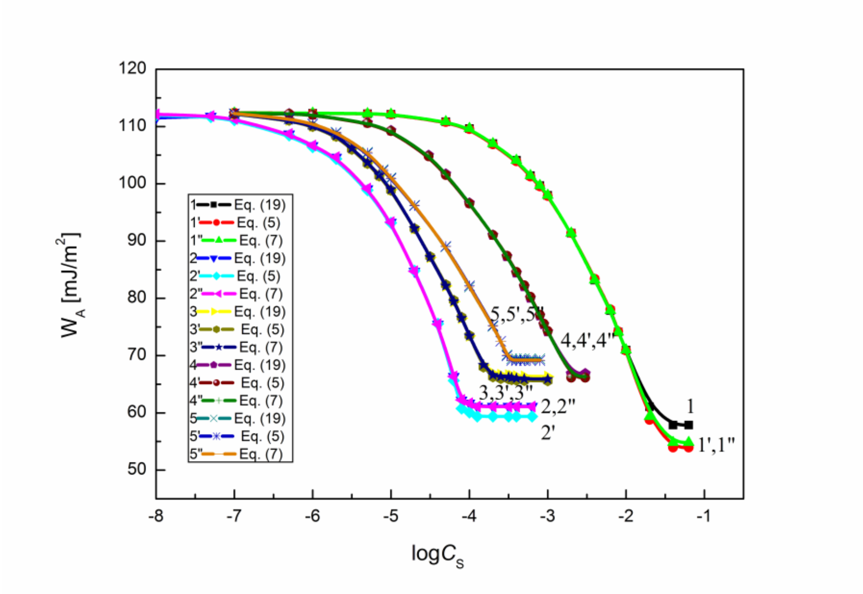

Fig. S26. A plot of the work of adhesion (W_A) of aqueous solution of OGP (curve 1, 1' and 1"), DDGP (curve 2, 2' and 2"), DM (curve 3, 3' and 3"), SMD (curve 4, 4' and 4") and SML (curve 4, 5' and 5") to the quartz surface vs. the logarithm of surfactant concentration ($\log C_S$). Curves 1 – 5, curves 1' – 5' and curves 1"-5" correspond to the W_A values calculated from Eq. (19), Eq. (5) and Eq. (7), respectively.

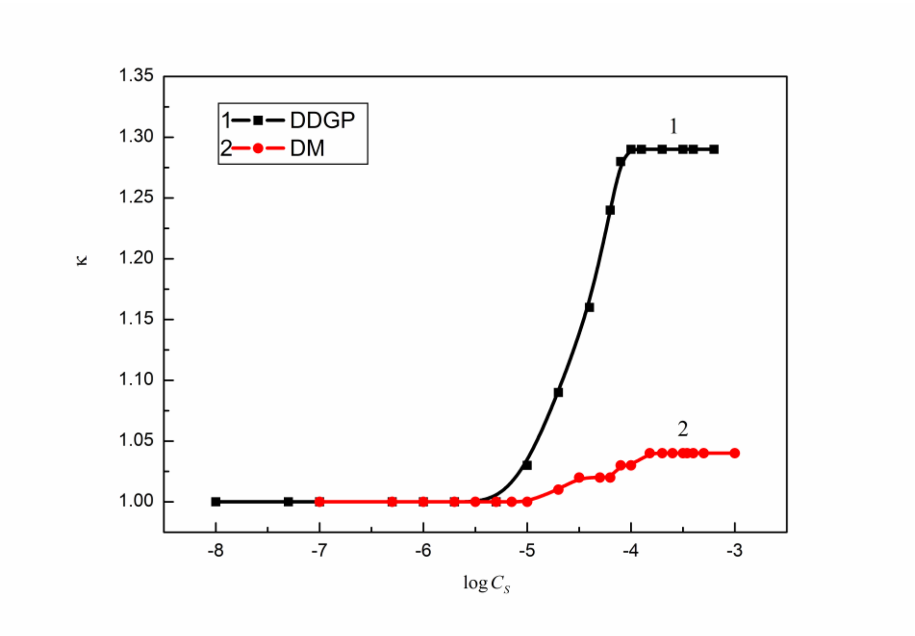

Fig. S27. A plot of the κ values calculated from Eq. (23) for DDGP (curve 1) and DM (curve 2) vs. the logarithm of their concentration (C_S).

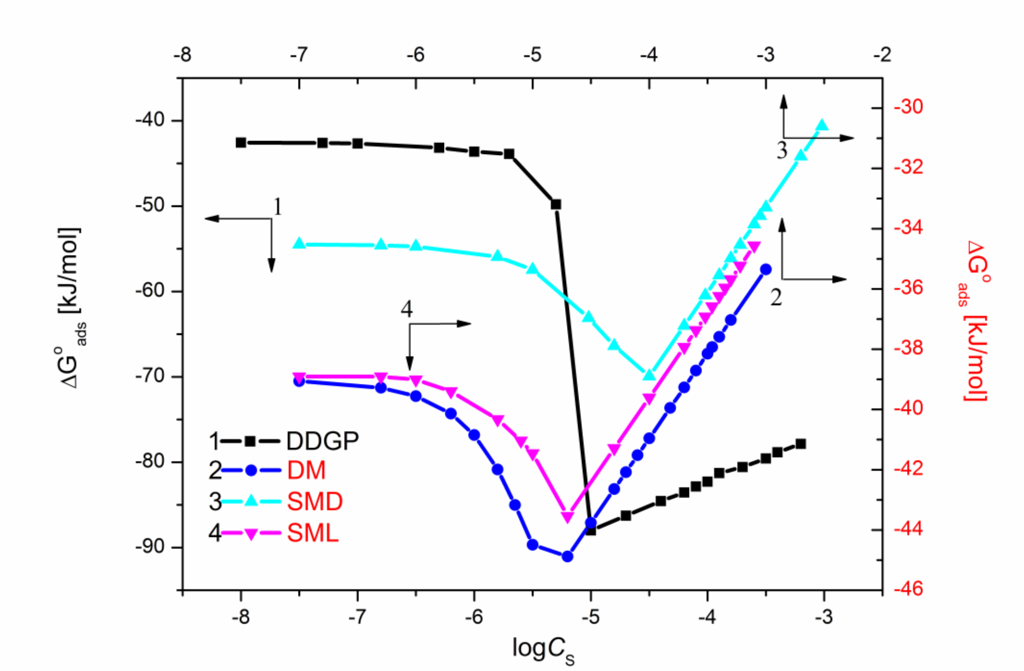

Fig. 28. A plot of the standard Gibbs free energy of adsorption (ΔG^o_{ads}) of DDGP (curve 1), DM (curve 2), SMD (curve 3) and SML (curve 4) at the PTFE-water interface at vs. the logarithm of surfactant concentration ($\log C_S$) calculated from Eq. (24).

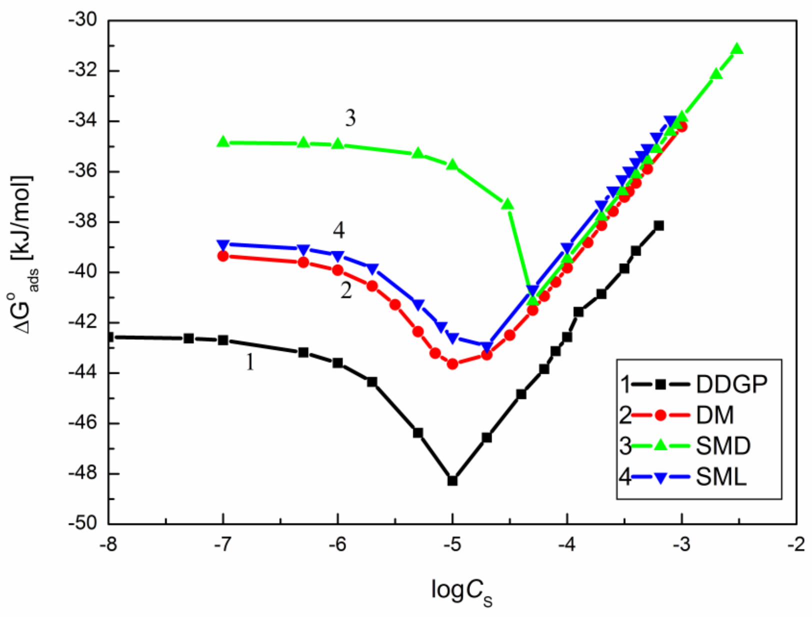

Fig. 29. A plot of the standard Gibbs free energy of adsorption (ΔG^{o}_{ads}) of DDGP (curve 1), DM (curve 2), SMD (curve 3) and SML (curve 4) at the PE-water interface at vs. the logarithm of surfactant concentration ($\log C_S$) calculated from Eq. (24).

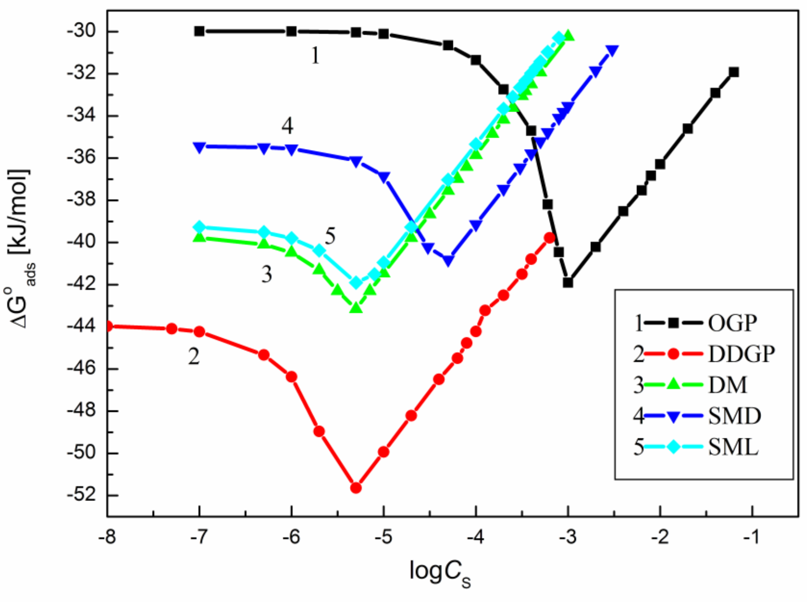

Fig. 30. A plot of the standard Gibbs free energy of adsorption (ΔG^{o}_{ads}) of OGP (curve 1), DDGP (curve 2), DM (curve 3), SMD (curve 4) and SML (curve 5) at the PMMA-water interface at vs. the logarithm of surfactant concentration ($\log C_S$) calculated from Eq. (24).

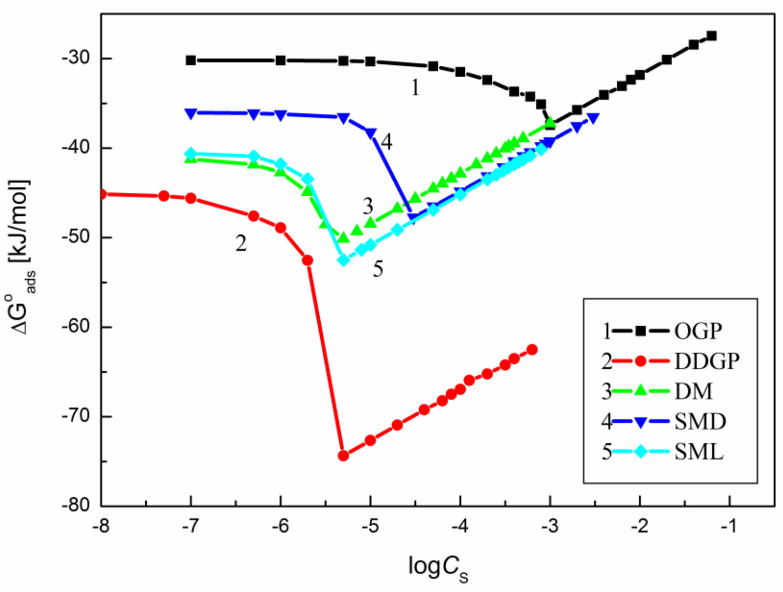

Fig. 31. A plot of the standard Gibbs free energy of adsorption (ΔG^{o}_{ads}) of OGP (curve 1), DDGP (curve 2), DM (curve 3), SMD (curve 4) and SML (curve 5) at the nylon 6-water interface at vs. the logarithm of surfactant concentration ($\log C_S$) calculated from Eq. (24).

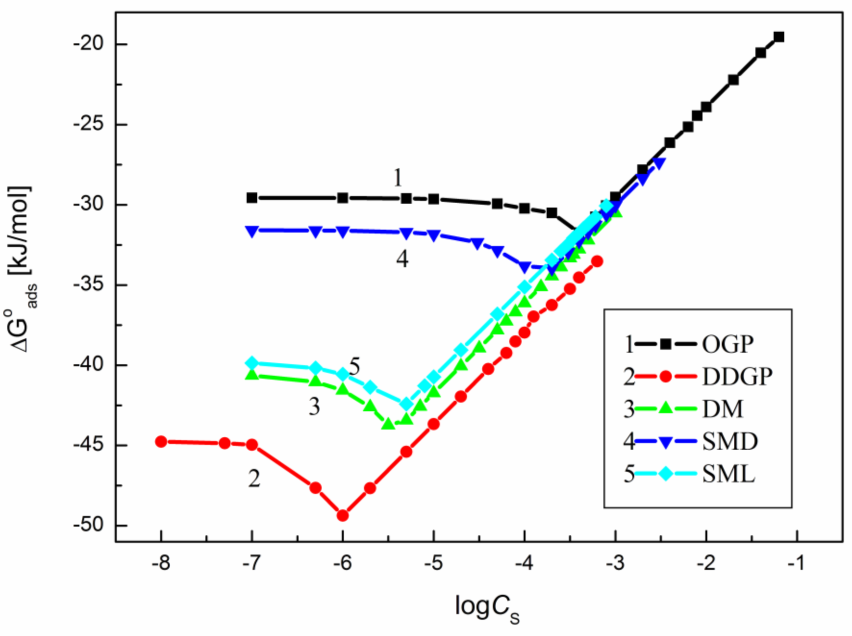

Fig. 32. A plot of the standard Gibbs free energy of adsorption (ΔG^{o}_{ads}) of OGP (curve 1), DDGP (curve 2), DM (curve 3), SMD (curve 4) and SML (curve 5) at the quartz-water interface at vs. the logarithm of surfactant concentration ($\log C_S$) calculated from Eq. (24).
